# Spontaneous Appearance of Triiodide Covering the Topmost Layer of the Iodide Solution Interface Without Photo-Oxidation

**DOI:** 10.1021/acs.est.3c08243

**Published:** 2024-02-14

**Authors:** Takakazu Seki, Chun-Chieh Yu, Kuo-Yang Chiang, Xiaoqing Yu, Shumei Sun, Mischa Bonn, Yuki Nagata

**Affiliations:** † Max Planck Institute for Polymer Research, Ackermannweg 10, 55128 Mainz, Germany; ‡ Graduate School of Science and Technology, Hirosaki University, Hirosaki, Aomori 036-8561, Japan; § Department of Physics, Applied Optics Beijing Area Major Laboratory, Beijing Normal University, Beijing 100875, China

**Keywords:** iodide, triiodide, surface propensity, sum-frequency generation spectroscopy, vibrational spectroscopy, vapor–water interface

## Abstract

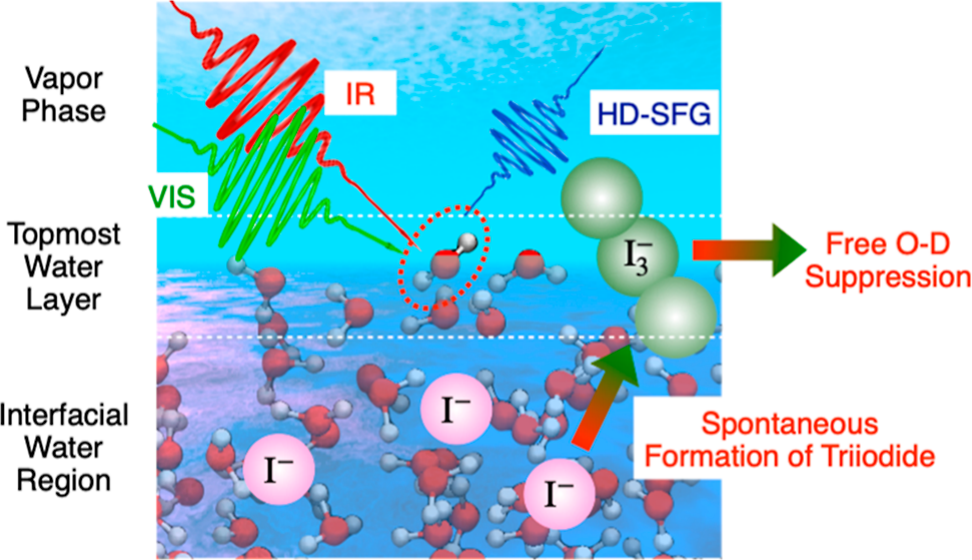

Ions containing iodine atoms at the vapor-aqueous solution interfaces critically affect aerosol growth and atmospheric chemistry due to their complex chemical nature and multivalency. While the surface propensity of iodide ions has been intensely discussed in the context of the Hofmeister series, the stability of various ions containing iodine atoms at the vapor–water interface has been debated. Here, we combine surface-specific sum-frequency generation (SFG) vibrational spectroscopy with *ab initio* molecular dynamics simulations to examine the extent to which iodide ions cover the aqueous surface. The SFG probe of the free O–D stretch mode of heavy water indicates that the free O–D group density decreases drastically at the interface when the bulk NaI concentration exceeds ∼2 M. The decrease in the free O–D group density is attributed to the spontaneous appearance of triiodide that covers the topmost interface rather than to the surface adsorption of iodide. This finding demonstrates that iodide is not surface-active, yet the highly surface-active triiodide is generated spontaneously at the water–air interface, even under dark and oxygen-free conditions. Our study provides an important first step toward clarifying iodine chemistry and pathways for aerosol formation.

## Abstract 2

Combined surface-specific spectroscopy and simulations demonstrate that iodide spontaneously forms extremely surface-active triiodide at the N_2_ gas–water interface even under dark and oxygen-free conditions, clarifying the surface composition of the iodide solution and suggesting an iodine-emission pathway from the seawater.

## Introduction

Halide ions at the vapor-aqueous solution interfaces play an essential role in aerosol nucleation and atmospheric chemistry. Among various halide ions, iodine exhibits appreciably more complex chemical behavior by varying its oxidation number from +5 to −1 and showing the lowest electron affinity.^1^ Iodide (I^–^) can be converted to triiodide (I_3_^–^) and iodate (IO_3_^–^),^2,3^ leading to polyvalent iodine compounds.^4^ Polyvalent iodine compounds at aqueous interfaces play a critical role in the nucleation of aerosol particles and the emission of iodine into the atmosphere. For example, iodine oxide and iodate allow for the formation of ultrafine aerosol particles with diameters of 3–10 nm,^5^ affecting cloud properties.^6−8^ Iodine species with different oxidation degrees undergo different reaction pathways toward iodine emission.^5,8−11^ In particular, the conversion of I^–^ into I_2_ in the absence of an oxidizing agent has been discussed in the past, while it is debatable.^2,12^ Unveiling the stability and surface propensity of various iodine derivatives at the vapor–water interface is essential for understanding the formation process and lifetime of aerosol particles.

Various theoretical and experimental techniques have been used to investigate the surface of the iodide salt solutions. Theoretically, molecular dynamics (MD) simulations have examined the surface propensity of halide ions;^13−22^ ions with a large radius tend to have a significant surface propensity, while the predicted surface propensity depends highly on the accuracy of the force field models. The surface of the aqueous iodide solutions, such as NaI and KI solutions, has been probed with interface-specific spectroscopy, including sum-frequency generation (SFG), second-harmonic generation,^23−31^ (photo)electron spectroscopy,^32−38^ and mass spectrometry.^39^ However, such theoretical and experimental studies have assumed that only iodide is present at the vapor–water interfaces when an iodide salt solution is prepared, despite the many polyatomic iodine compounds that have been proposed to be present at interfaces.^3,5,40^ The following question thus arises: What is the iodine species that is stably present and covers the topmost layer of the NaI solution in the nitrogen and dark environment?

To address these questions, we probe the N_2_ gas-D_2_O solution at the NaI interface by combining polarization-dependent heterodyne-detected SFG (PD-HD-SFG) with *ab initio* MDs (AIMD) simulations. The PD-HD-SFG technique^41^ allows us to unambiguously quantify the density of free, non-hydrogen-bonded O–D groups at the interface by accounting for possible changes in the molecular orientation.^42,43^ This is achieved by measuring the imaginary part of the complex-valued second-order susceptibility (χ_eff_^(2)^) for different polarization combinations.^44^ Since standard force field simulations can misrepresent the ion–water interaction,^45^ AIMD simulations at the *ab initio* level of theory are required to simulate the free O–D behavior and the stability of ions at aqueous interfaces.

Here, we show that the free O–D population is drastically reduced with increasing NaI concentration ([NaI]) in D_2_O solution, but only when [NaI] > 2 M. AIMD simulation reveals that the drastic reduction of the free O–D population cannot arise from interfacial iodide adsorption but rather can be attributed to the adsorption of triiodide ions. We further infer that the concentration of triiodide at the interface is ∼10^8^ times higher than that in the bulk. We discuss the interfacial chemical equilibrium and its implications for atmospheric chemistry.

## Results and Discussion

**Figure 1:**
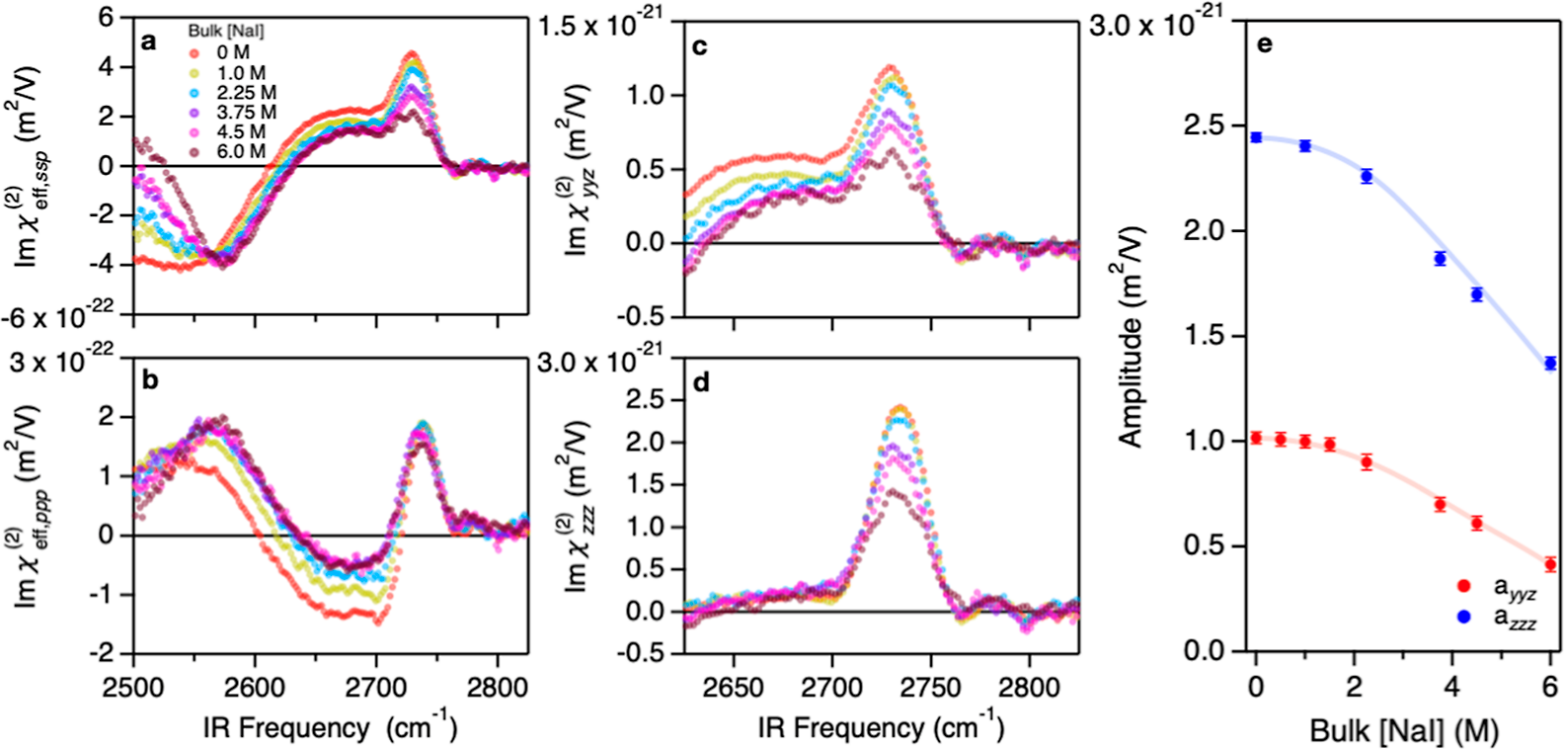
PD-HD-SFG measurement at the N_2_ gas-D_2_O interface. (a) Imχ_eff,ssp_^(2)^ and (b) Imχ_eff,ppp_^(2)^ spectra were obtained at the N_2_ gas-D_2_O interface in the absence and presence of NaI. Absolute amplitudes were obtained using the known effective surface nonlinear susceptibility of *z*-cut *a*-quartz (see Supporting Information). (c) Imχ_*yyz*_^(2)^ and (d) Imχ_*zzz*_^(2)^ spectra after correction for the Fresnel coefficients. The error bars from multiple measurements of these spectra are given in Figure S1. (e) Variations of the free O–D peak amplitudes, *a*_*yyz*_ and *a*_*zzz*_, obtained from fits to the Imχ_*yyz*_^(2)^ and Imχ_*zzz*_^(2)^ spectra, respectively (see Supporting Information). Lines serve to guide the eyes.

Figure 1a and 1b display the measured Imχ_eff,ssp_^(2)^ and Imχ_eff,ppp_^(2)^ spectra at the N_2_ gas-D_2_O solution interface with varying [NaI], respectively. The Imχ_eff,ssp_^(2)^ spectrum at the N_2_ gas-neat D_2_O interface shows the 2730 cm^–1^ positive peak, 2650 cm^–1^ positive shoulder peak, and 2550 cm^–1^ negative band, consistent with the previous study.^46^ The 2730 cm^–1^ peak, the 2650 cm^–1^ shoulder peak, and the 2550 cm^–1^ band originate from free O–D groups, the antisymmetric O–D stretch mode of the D_2_O molecules donating two hydrogen bonds, and hydrogen-bonded O–D groups, respectively.^47−49^ Upon the addition of NaI, the Imχ_eff,ssp_^(2)^ amplitude decreases above 2560 cm^–1^, while it increases below 2560 cm^–1^, consistent with refs (29 and 50). The Imχ_eff,ppp_^(2)^ spectra commonly show a 2730 cm^–1^ positive peak, a 2650 cm^–1^ negative band, and a broad 2550 cm^–1^ positive band. Upon increasing [NaI], the 2730 cm^–1^ contribution does not change significantly, unlike in the Imχ_eff,ssp_^(2)^ spectra, while the peak amplitude below 2720 cm^–1^ decreases.

From the χ_eff,ssp_^(2)^ and χ_eff,ppp_^(2)^ spectra, we obtain the *yyz* and *zzz* components of the second-order susceptibilities, χ_*yyz*_^(2)^ and χ_*zzz*_^(2)^ (see Supporting Information),^51^ where the *xz*-plane forms the incident plane and the *z*-axis forms the surface normal. Because we are primarily interested in quantifying the response of the free O–D groups on the surface (rather than that of the hydrogen-bonded O–D groups below the surface), the relevant Fresnel factors were calculated using the interfacial refractive index deduced from the simulations in a similar manner to refs (52–54) for the free O–D groups’ interfacial location (see Supporting Information Section 3.2). The inferred Imχ_*yyz*_^(2)^ and Imχ_*zzz*_^(2)^ spectra are displayed in Figure 1c and 1d, respectively. Both spectra show that the free features of the elongated O–D feature diminish with increasing [NaI]. A closer look at the peak amplitude extracted from the fits (Figure 1e) reveals a highly nonlinear behavior, also apparent from the raw data: the Imχ_*yyz*_^(2)^ and Imχ_*zzz*_^(2)^ amplitudes of the free O–D peak (*a*_*yyz*_ and *a*_*zzz*_, respectively) only start to decrease above [NaI] = ∼2 M. The surface pressure data of the NaI solution also shows a similar nonlinear behavior above ∼2 M (see Supporting Information). In contrast, we could not see such a nonlinear trend for other halide ions, such as Cl^–^, as displayed in Supporting Information.^50^

**Figure 2:**
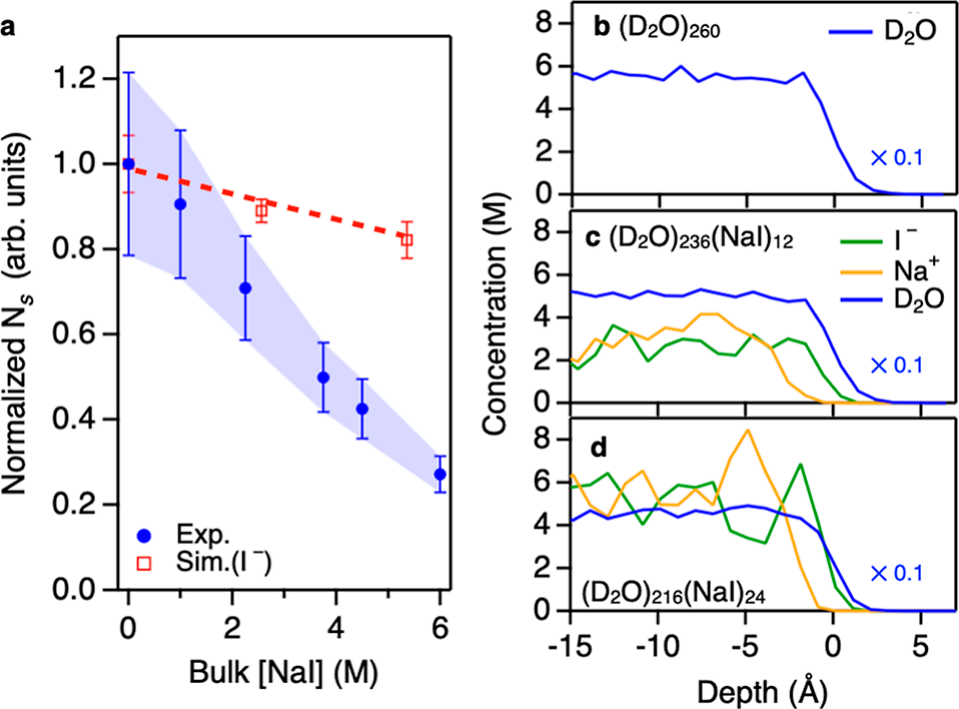
Surface population of the free O–D chromophores as a function of [NaI] in bulk. (a) Normalized surface density of the free O–D chromophores *N*_S_ as a function of [NaI]. *N*_S_ values are normalized to the value for the bare surface. The shaded region around the experimental data represents the error bars obtained from the fits and subsequent orientational analysis (Supporting Information); the error bars on the simulation data represent the 95% confidence intervals obtained from multiple data sets. The red dotted line shows the weak variation of the free O–D population expected from AIMD simulations. (b–d) Computed interfacial concentration profiles at the vapor–water interface in the absence (b) and presence of NaI (c,d). The water concentration profiles are rescaled by a factor of 0.1. Zero depth is the position of the Gibbs dividing surface for water.

Since the SFG peak amplitude of the free O–D peak is determined not only by the surface density of the free O–D stretch chromophores (*N*_S_) but also by the orientation of the free O–D group,^51,55^ one needs to disentangle these contributions to access *N*_S_. To do so, we extracted the free O–D peak area in the Imχ_*yyz*_^(2)^ and Imχ_*zzz*_^(2)^ spectra (denoted as *A*_*yyz*_ and *A*_*zzz*_, respectively). Subsequently, we obtained *N*_S_ using the following relations^56^
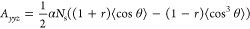
1
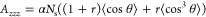
2where θ denotes the average angle of the chromophores with respect to the surface normal, *α* is the hyperpolarizability, and *r* is the depolarization ratio of the free O–D stretch mode. We used *r* = 0.15.^57^ By using an exponentially decay-shaped function for the orientational distribution^57^ and assuming the rotational motion to be slow compared to vibrational energy relaxation,^43^ we obtained *N*_S_ as a function of [NaI], as displayed in Figure 2a. This figure indicates that *N*_S_ decreases drastically with increasing [NaI], particularly at elevated [NaI]. The decrease in *N*_S_ results from the reduction of the contact area of the N_2_ gas–water interface due to increasing the number of ions at the interface. Note that since the change in the peak area *A*_*yyz*_ arises not only from the change in *N*_S_ upon the variation of [NaI] but also from the change of the orientation of the free O–D group, as is discussed in Supporting Information Section S3.4, the careful separation of the contributions from the variation of ⟨cosθ⟩ and ⟨cos^3^θ⟩ and from *N*_S_ through the PD-HD-SFG was needed.

Iodide (I^–^) has long been suggested to be a species with a high surface propensity.^13,15,16,23^ Can the reduction of *N*_*S*_ be rationalized by the appearance of iodide in the topmost water layer? To answer this question, we carried out AIMD simulations at the vapor-D_2_O interface ((D_2_O)_260_) and vapor-D_2_O solutions of NaI, (D_2_O)_236_(NaI)_12_, and (D_2_O)_216_(NaI)_24_, and we computed the variations of *N*_*S*_ of the free O–D group from the AIMD trajectories. The simulation details can be found in the Supporting Information. The obtained *N*_S_ vs [NaI] data is plotted in Figure 2a, where the values of [NaI] were obtained from the interfacial concentration profiles (Figure 2b–d) in the *z* < −7 Å region (bulk region). Note that the origin point of the *z*-axis is the position of the Gibbs dividing surface. The discrepancy between the experiment and simulation illustrates that the presence of iodide alone cannot explain the substantial reduction of *N*_S_ observed in the experiment. In fact, the concentration profiles in Figure 2b–d show that the I^–^ density at the interface (*z* ∼ 0 Å) is comparable with that in the bulk (*z* < −7 Å) at all concentrations. This means that the iodide is not particularly surface-active, and the reduction of the contact area of the vapor–water interface due to the iodide is limited. Note that this trend differs from the previous classical MD simulations^13−16,18^ but is consistent with another AIMD simulation,^21^ indicating that force field parameters may erroneously lead to an apparent stabilization of I^–^ at the vapor–water interface. This notion is consistent with the recent finding that the repulsive force of the Lennard-Jones potential,^45^ together with the underestimated dielectric property of simulated water,^58,59^ erroneously predicts ions to be surface active.

Clearly, the surface activity of the iodide ion cannot explain our observations. What moiety is formed and appears at the N_2_ gas–water interface, reducing the free O–D group population (*N*_S_)? A plausible candidate is a polyvalent iodine ion such as IO^–^, IO_2_^–^, IO_3_^–^, and I_3_^–^. Among these species, we can rule out IO^–^, IO_2_^–^, and IO_3_^–^ as the surface active species since these ions are more hydrophilic than iodide and thus will be located in the bulk of the water. Indeed, a recent AIMD study^60^ has shown that all the atoms of the IO_3_^–^ ion can form strong hydrogen bonds with water molecules, implying that the surface propensity of IO_3_^–^ ions would be limited. We also measured the Imχ_*yyz*_^(2)^ spectra from the surface of a NaIO_3_ solution and found that, indeed, the free O–D peak feature is unchanged upon the addition of NaIO_3_ (see Figure S5), confirming that IO_3_^–^ is not a species to change *N*_S_ of the free O–D groups.

**Figure 3:**
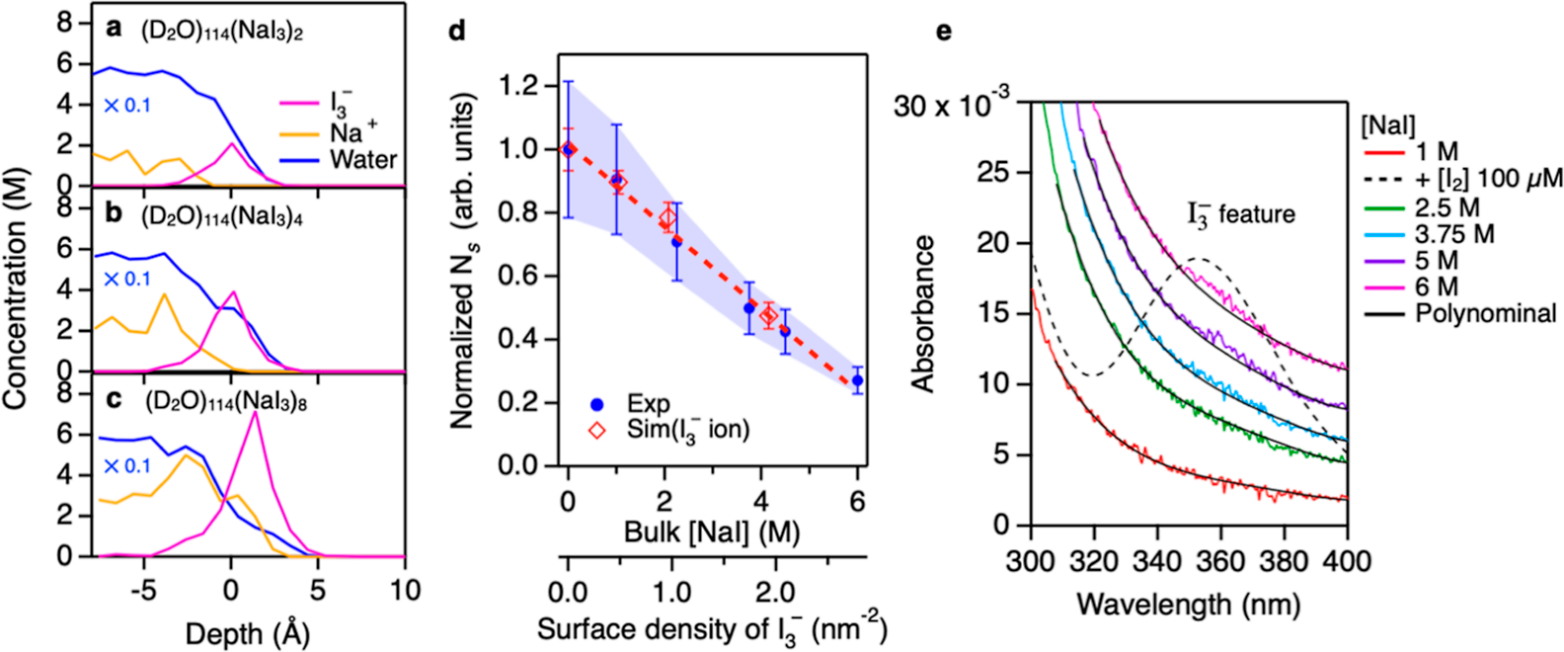
Variation of the surface density of the free O–D chromophores in the presence of I_3_^–^. (a–c) Interfacial concentration profiles for NaI_3_ solutions with various concentrations. The water concentration profile was rescaled by a factor of 0.1 for clarity. (d) Comparison of the variations of free O–D populations between the simulation and experiment. The experimental data are identical to the data in Figure 2a. The surface density of I_3_^–^ for the experimental data was estimated from the UV–vis spectra and AIMD simulation (see Supporting Information Sections S7 and S8). The red dotted line serves to guide the eyes. (e) UV–vis spectra for NaI samples with varying bulk concentrations of NaI, together with the absorption spectrum for the 100 μM I_3_^–^ ion (dotted line). The absorption spectrum of I_3_^–^ was scaled by a factor of 0.01. The black solid lines represent the baseline computed based on the fifth-order polynomial function to clarify the appearance of the 350 nm feature.

After excluding IO_3_^–^ as a species that reduces *N*_S_, we explored the impact of I_3_^–^ on *N*_S_ using AIMD simulations. The interfacial concentration profiles at the N_2_ gas-D_2_O solution of NaI_3_ interfaces with various NaI_3_ concentrations are plotted in Figure 3a–c, while the variation of *N*_S_ is plotted in Figure 3d. The interfacial concentration profiles reveal an extremely high surface propensity of I_3_^–^ at the N_2_ gas–water interface. Such a high propensity reduces *N*_S_, as is evident from Figure 3d; when the surface density of I_3_^–^ is 2.0 nm^–2^, *N*_S_ decreases by ∼60%. This indicates that the contact area of the N_2_ gas–water interface can be reduced due to the presence of I_3_^–^. The higher surface propensity of I_3_^–^ than I^–^ stems from a much lower negative charge density of I_3_^–^ than that of I^–^.^61^

The interfacial concentration profiles obtained from the AIMD simulation suggest that [NaI_3_] is high at the interfaces but is extremely low in the bulk. This implies that any I_3_^–^ present in solution will appear at the surface, and trace amounts of I_3_^–^ are sufficient to modify the surface density of the free O–D groups. To examine whether any I_3_^–^ is present in our samples, we measured bulk ultraviolet–visible (UV–vis) spectra of the NaI–H_2_O solutions with various [NaI]. The data are displayed in Figure 3e. A 350 nm feature, a fingerprint of the I_3_^–^ species, is not detectable for [NaI] < 4 M. For [NaI] > 4 M, a tiny 350 nm feature is apparent. From the UV–vis data, we infer an upper limit of I_3_^–^ concentration to be ∼47 and ∼54 nM at [NaI] = 5 and 6 M, respectively.^62^ The AIMD simulation indicates that such extremely low concentrations of I_3_^–^ in the bulk are sufficient to modify the water surface since, in the simulations, the density of I_3_^–^ is near-zero in the *z* < –5 Å region (bulk region). Furthermore, by computing the energy difference between the partially hydrated and fully hydrated states of I_3_^–^, we infer the population difference between the interface and bulk to be different by a factor of 3 × 10^8^ (see Supporting Information Section S8) to predict the surface density of I_3_^–^ at the corresponding [NaI] in bulk (Figure 3d). As is clear from the quantitative comparison in Figure 3d, the AIMD simulation of NaI_3_ captures the trend from the experiment.

To confirm that the I_3_^–^ species critically affects the free O–D peak amplitude, we compared Imχ_eff,ssp_^(2)^ spectra from the surface of a 1.5 M NaI solution, a 1.5 M NaI + 1 μM I_2_ solution, and a 1.5 M NaI + 1 μM I_2_ + 100 mM Na_2_S_2_O_3_ solution. If the reduction of the free O–D peak arises from I_3_^–^, the addition of I_2_ to the NaI solution should lead to the reduction of the free O–D peak via the generation of I_3_^–^^62^
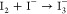
3

In contrast, Na_2_S_2_O_3_ is known to serve as a strong reductant. Thus, the addition of Na_2_S_2_O_3_ to the solution containing I_3_^–^ leads to the following chemical reaction
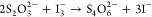
4

Because I_3_^–^ is reduced, one can expect that the free O–D peak for the 1.5 M NaI + 1 μM I_2_ + 100 mM Na_2_S_2_O_3_ solution is comparable to the free O–D peak for the 1.5 M NaI solution.

**Figure 4:**
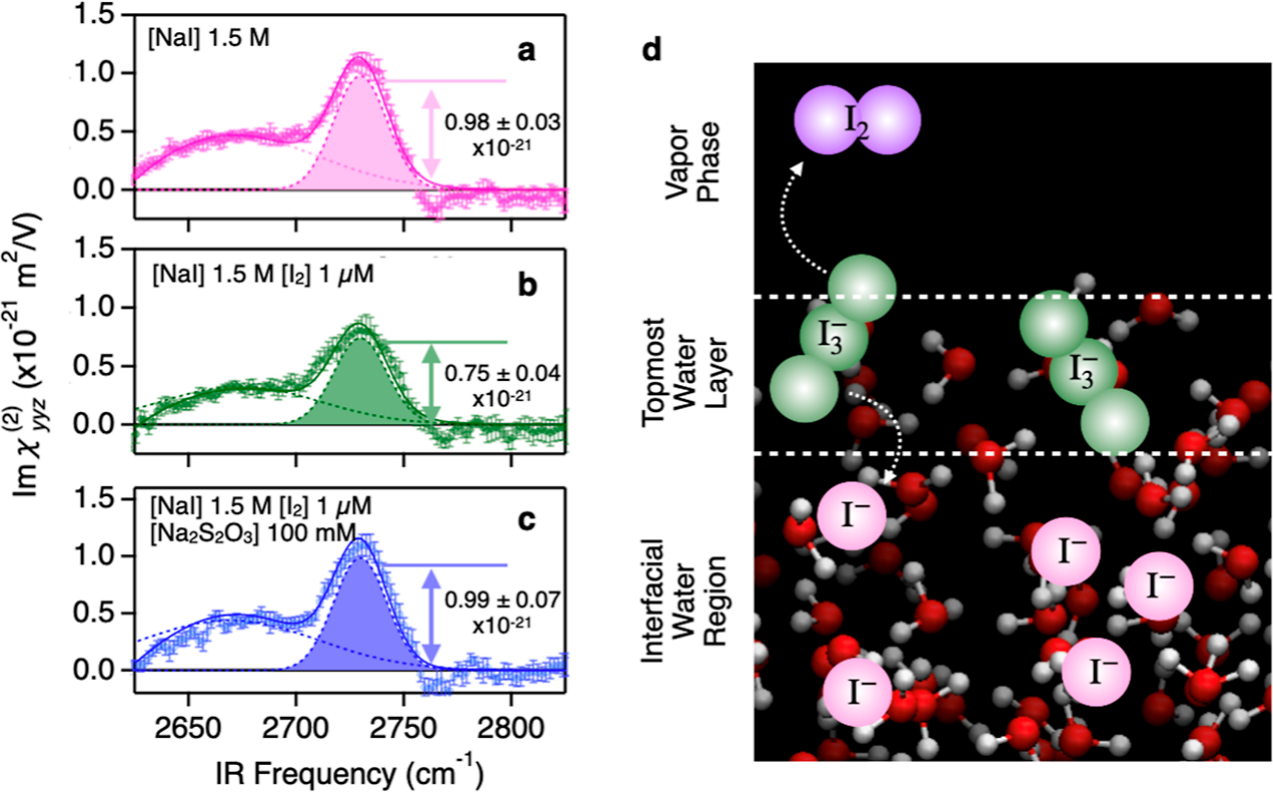
Effect of chemically generated I_3_^–^ on the free O–D amplitude. (a–c), Imχ_*yyz*_^(2)^ spectra at the N_2_ gas-D_2_O solutions interfaces: 1.5 M NaI D_2_O solution (a), 1.5 M NaI and 1 μM I_2_ D_2_O solution (b), and 1.5 M NaI, 1 μM I_2,_ and 100 mM Na_2_S_2_O_3_ D_2_O solution (c). The solid lines represent the fits, and the dotted lines indicate the peak contributions obtained from the fits. The free O–D peak feature is highlighted by filling in the dotted lines. The arrows show the amplitude of the free O–D peak. (d) Schematic representation of the ion organization at the vapor-aqueous solution of the NaI interface. I^–^ does not perturb the topmost water structure and thus is present below the topmost water layer. On the other hand, I_3_^–^ is present in the topmost water layer due to its high surface propensity. I_3_^–^ can serve as a reservoir/emitter of I_2_ at the interface via the reaction I^–^ + I_2_ ⇄ I_3_^–^.^62^ The error bars indicate the 95% confidence intervals of the multiple measurements.

The Imχ_*yyz*_^(2)^ spectra obtained from the measured Imχ_eff,ssp_^(2)^ spectra are displayed in Figure 4a–c. The Gaussian fit to the spectra (Supporting Information) provides the amplitude of the free O–D peak. The amplitude for the NaI solution is (0.98 ± 0.03) × 10^–21^ m^2^/V, while it decreases to (0.75 ± 0.04) × 10^–21^ m^2^/V upon the addition of I_2_. By reducing the I_2_-containing sample using Na_2_S_2_O_3_, the amplitude was recovered to (0.99 ± 0.07) × 10^–21^ m^2^/V, i.e., equal to the amplitude of pure NaI solution within experimental uncertainty. Note that this recovery took more than 1 h, presumably because of the low surface activity of Na_2_S_2_O_3_. This observation is consistent with the scenario described above, manifesting that the free O–D reduction arises from I_3_^–^ and not from I^–^ (Figure 4d).

Our data witness the extreme surface propensity of I_3_^–^ at the N_2_ gas–water interface and the absence of I^–^ at the topmost N_2_ gas–water interface. This appearance of I_3_^–^ occurs via the salting-out of large hydrophobic ions (I_3_^–^) by small hydrophilic ions (I^–^).^63^ Such insights into the ion composition and distribution in aerosols are crucial because they determine the chemistry occurring inside and on the surface of aerosols. This chemistry also depends on the aerosol size, which governs the ratio of surface area to the number of I_3_^–^, a molecular reservoir of I_2._ Since I_3_^–^ is localized in the outermost aerosol layer, it can emit I_2_ into the vapor phase via the backward reaction (Figure 4d). This chemical pathway may contribute to I_2_ emission into the atmosphere, in addition to the well-known I_2_ emission from I^–^ and HOI.^3,64^

Finally, we note other experimental techniques that can shed light on this challenge. Second harmonic generation spectroscopy^65^ and electronic SFG^66−68^ may be able to probe I_3_^–^ if the visible beam wavelength is set to match the optical transition of I_3_^–^ and the Raman cross section is sufficiently large. Probing different iodine species across a wide wavelength region may clarify the iodine chemistry at the interface. Liquid jet X-ray photoelectron spectroscopy is another powerful tool to probe surface composition, but this technique may give different results compared to this SFG study, as in the liquid jet, the surface is generated quasi-instantaneously, and we expect the generation of I_3_^–^ to be slower than the time scale of the surface generation in these types of experiments.^35,38^

## Environmental Implications

Here, by combining PD-HD-SFG spectroscopy with AIMD simulation, we confirmed that the iodide ion does not significantly reduce the contact area of the N_2_ gas–water interface; iodide ions are instead located below the topmost water layer. When the iodide ion concentration increases, the triiodide ions are more present at the vapor–water interface, resulting in a ∼10^8^ times higher concentration at the surface than in bulk. Such salting-out of hydrophobic ions by other hydrophilic ions has been universally observed.^63,69−72^ Surprisingly, triiodide appears spontaneously at the interface in a dark and nitrogen atmosphere, which is in line with the recent report by Guo et al.^12^ While >1 M concentration of iodide is unlikely in the bulk solution in nature, an extremely high local surface concentration of the hydrophobic triiodide ions at the aqueous interfaces is known to be enabled by the salting-out of these ions by other hydrophilic ions.^63,69−72^ This suggests that the vapor–water interface provides a unique sink and reaction environment for triiodide ions. Our result demonstrates that the reaction mechanism and stability of the polyatomic iodine differ significantly between the bulk and at the interface. Together with the salting-out of triiodide by other ion species in the sea, our current study indicates that triiodide can be present on the surfaces even without sunlight (at night).

### Experimental and Simulation Methods

#### Sample Preparation

Sodium iodide (>99.5%) was purchased from Alfa Aesar. Sodium iodate (>99.5%), iodine (>99.99%), and D_2_O (>99.9%) were obtained from Sigma-Aldrich. Sodium thiosulfate (≥99%, anhydrous) was obtained from Carl Roth. These materials were used as received. We used H_2_O obtained from a Milli-Q machine (a resistance of 18.2 MΩ cm). To avoid oxidation of the iodide ion as much as possible, we dissolved sodium iodide salt into D_2_O (H_2_O) under an N_2_ atmosphere and in a dark room just before SFG (UV–visible absorption) experiments. We poured the resultant D_2_O solution of NaI into a PTFE dish with a diameter of 6 cm and measured the SFG spectra from the samples. We emphasize that we avoided the laser irradiation for the samples before SFG spectra measurements. For generating I_3_^–^ ions, we dissolved I_3_^–^ (50 mg) into NaI solution (1.5 M, 20 mL). The concentration of the generated I_3_^–^ ion was calculated to be ∼10 mM based on the equilibrium constant of ∼700 M^–1^ for the triiodide formation reaction of I^–^ + I_2_ ⇄ I_3_^–^.^73^ By diluting this solution with a 1.5 M NaI solution, we obtained a 1.5 M NaI solution with 1 μM I_2_ and used it in Figure 4b. For NaI solution with 1 μM I_2_ and 100 mM Na_2_S_2_O_3_, we first dissolved I_2_ (50 mg) into Na_2_S_2_O_3_ solution (100 mM, 20 mL) to exclude the possibility that a trace amount of I_2_ remained in the solution. After that, we diluted this solution with a NaI and Na_2_S_2_O_3_ mixture solution to have 1 μM I_2_ and used it in Figure 4c. Note that the sequential addition of Na_2_S_2_O_3_ into the NaI solution with 1 μM I_2_ gave a similar trend in the recovery of the free O–D contribution.

#### HD-SFG Measurement

The details of our PD-HD-SFG setup are presented in ref (74). Briefly, we focused the infrared (IR) and visible beams collinearly onto a *y*-cut quartz to generate a local oscillator (LO) signal. A 5 mm-thick SrTiO_3_ plate was inserted into the beam path to generate the delay for the LO beam relative to the other beams. These beams were refocused onto the N_2_ gas-D_2_O solution at the NaI interface. The angles of incidence were set to 45° with respect to the surface normal. We used *ssp* and *ppp* polarization combinations, where *ssp* (*ppp*) denotes *s*- (*p*-)polarized SFG, *s*- (*p*-)polarized visible, and *p*- (*p*-)polarized IR beams. The measurements were performed under a nitrogen atmosphere with a humidity of less than 0.1%. After 15 min of equilibration of the samples under a nitrogen atmosphere, the HD-SFG measurements were carried out. The exposure time of the measurement was set to three min, and the data obtained was averaged over 15 min. Note that, over the course of the measurements, we did not observe changes in the spectral feature within this time scale. Freshly prepared NaI solutions were used to prevent oxidation due to oxygen in the air. Further details of the setup and HD-SFG measurements are present in the Supporting Information.

#### AIMDs Simulation

AIMD simulations for the systems of pure D_2_O, NaI in D_2_O, and NaI_3_ in D_2_O have been conducted with the CP2K code.^75^ The system of pure D_2_O consisted of 260 D_2_O molecules, while the systems of NaI in D_2_O consisted of 216 D_2_O and 24 NaI [(D_2_O)_216_(NaI)_24_] and 236 D_2_O and 12 NaI [(D_2_O)_236_(NaI)_12_]. These molecules were contained in the 14.4 × 14.4 × 70.0 Å cell. The systems of NaI_3_ in D_2_O consisted of 114 D_2_O and 8 NaI_3_ [(D_2_O)_114_(NaI_3_)_8_], 114 D_2_O and 4 NaI_3_ [(D_2_O)_114_(NaI_3_)_4_], and 114 D_2_O and 2 NaI_3_ [(D_2_O)_114_(NaI_3_)_2_]. These molecules were contained in the 14.4 × 14.4 × 50.0 Å cell. For the pure D_2_O and NaI in D_2_O systems, we prepared the 5 random structures for the pure D_2_O system and the 10 random structures for the NaI in D_2_O and NaI_3_ in D_2_O systems using the Packmol code.^76^ After 1 ns of equilibration in the force field MD simulation, we performed an AIMD simulation for these samples. Further details of the procedure can be found in the Supporting Information.

## Supporting Information Available

Details of AIMD simulation; definition of the free O–D group for NaI and NaI_3_ solutions; protocol for interfacial and bulk concentrations of I^–^ and I_3_^–^ ions; experimental details for HD-SFG setup and UV–visible absorption measurement; amplitude calibration of the HD-SFG spectra and Fresnel factor correction; procedures for the fits of HD-SFG spectra and for calculating the orientational and density analysis of the free O–D group; surface pressure data of NaCl and NaI solutions; comparison of NaBr and NaI samples; comparison of the neat D_2_O and NaIO_3_ samples; energy difference of the I_3_^–^ molecule at the water–air interface and in the bulk; and photosensitivity of the NaI sample under SFG measurements (PDF)
