# Supplementary material for: Spontaneous Appearance of Triiodide Covering the Topmost Layer of the Iodide Solution Interface Without Photo-Oxidation
Source: Environ Sci Technol. 2024 Feb 14;58(8):3830–7. doi: 10.1021/acs.est.3c08243 (PMC10902846; doi:10.1021/acs.est.3c08243)
Supplement: Supplementary file 1 — es3c08243_si_001.pdf [file es3c08243_si_001.pdf]

Supporting Information:

Spontaneous Appearance of Triiodide Covering the Topmost Layer of Iodide Solution Interface without Photo-Oxidation

Takakazu Seki,[1,2,§] Chun-Chieh Yu,[1,§] Kuo-Yang Chiang,[1] Xiaoqing Yu,[1] Shumei Sun[3], Mischa Bonn,[1,] and Yuki Nagata[1,*]*

1. Max Planck Institute for Polymer Research, Ackermannweg 10, 55128 Mainz, Germany
2. Graduate School of Science and Technology, Hirosaki University, Hirosaki 036-8561, Aomori, Japan
3. Department of Physics, Applied Optics Beijing Area Major Laboratory, Beijing Normal University, Beijing 100875, China

Corresponding author *: nagata@mpip-mainz.mpg.de, bonn@mpip-mainz.mpg.de

§T. S. and C.C.Y. contributed to this work equally.

This supporting information includes 30 pages, 4 tables, and 8 figures.

Contents

Section 1. Simulation protocols: *ab initio* molecular dynamics (AIMD) simulation, definition of the free O-D groups for NaI and NaI_3 samples, surface density of the free O-D groups, determination of bulk and interfacial concentrations of I^- and I_3^-.

Section 2. Experimental details: experimental setups for SFG measurement and UV-visible absorption measurement.

Section 3. Data processing and analysis: amplitude calibration of the SFG spectra at different polarizations, Fresnel factor correction, fits of SFG spectra, orientational and number density analysis.

Section 4. Surface pressure of NaCl and NaI aqueous solutions.

Section 5. The variation of the free O-H amplitude at NaBr aqueous solution interface.

Section 6. Comparison of the neat D_2O and $NaIO_3$ samples.

Section 7. Determination of bulk I_3^- concentration based on the UV-visible spectra.

Section 8. Energy difference of I_3^- molecule at the water-air interface and in the bulk.

Section 9. Photosensitivity of NaI Sample under SFG Measurements.

Table S1. The fitting parameters used for describing the $\mathrm{Im}\chi_{yyz}^{(2)}$ spectra for the N_2 gas-neat D_2O and N_2 gas-NaI aqueous solution interfaces.

Table S2. The fitting parameters used for describing the $\mathrm{Im}\chi_{zzz}^{(2)}$ spectra for the N_2 gas-neat D_2O and N_2 gas-NaI aqueous solution interfaces.

Table S3. The fitting parameters used for describing the $\mathrm{Im}\chi_{yyz}^{(2)}$ and $\mathrm{Im}\chi_{zzz}^{(2)}$ spectra obtained from the setup 2 for the N_2 gas-neat D_2O interface.

Table S4. Thermal averaged values and the surface density for the free O-D groups at the N_2 gas-water interface in the presence and absence of NaI.

Figure S1. $\mathrm{Im}\chi^{(2)}_{yyz}$ (a) and $\mathrm{Im}\chi^{(2)}_{zzz}$ (b) spectra after removal of the Fresnel coefficients.

Figure S2. $\mathrm{Im}\chi^{(2)}_{yyz}$ (a) and $\mathrm{Im}\chi^{(2)}_{zzz}$ (b) spectra for the entire fitting region for neat D_2O and NaI samples.

Figure S3. $\mathrm{Im}\chi^{(2)}_{yyz}$ and $\mathrm{Im}\chi^{(2)}_{zzz}$ spectra obtained from the setup 2 for the entire fitting region for neat D_2O.

Figure S4. (a) The ratio of A_{yyz}/A_{zzz} as a function of the average orientational angle of the free O-D groups. (b) The average angle of the free O-D group $\langle\theta\rangle$ and the normalized number of the free O-D chromophores N_s as a function of the NaI concentrations.

Figure S5. Surface pressure data of NaCl and NaI aqueous solutions as a function of salt concentrations at 25 °C.

Figure S6. (a) $\mathrm{Im}\chi^{(2)}_{yyz}$ spectra obtained from the N_2 gas-NaBr solution interface after removal of the Fresnel coefficients. (b) Variations of the 3700 cm^{-1} peak amplitude for NaBr samples and the 2730 cm^{-1} peak amplitude against the bulk concentrations of [NaBr] and [NaI], respectively.

Figure S7. Comparison of the $\mathrm{Im}\chi^{(2)}_{yyz}$ spectra for the neat D_2O and $NaIO_3$ samples.

Figure S8. (a) Time evolution of $\mathrm{Im}\chi^{(2)}_{\mathrm{eff},ssp}$ spectra for the N_2 gas-D_2O interface with [NaI] = 3.75 M. The legend indicates the elapsed time after starting the SFG measurement. (b) Comparison of the UV-visible spectra of NaI solution samples with [NaI] = 3.75 M with and without visible and IR laser irradiation for 1 hour.

1. Simulation protocols

1.1. *Ab initio* molecular dynamics (AIMD) simulation

AIMD simulations for the systems of pure D_2O, NaI in D_2O, and NaI_3 in D_2O have been conducted with the CP2K code.[1] The system of pure D_2O consisted of 260 D_2O molecules, while the systems of the NaI in D_2O consisted of 216 D_2O and 24 NaI ($(D_2O)_{216}(NaI)_{24}$) and 236 D_2O and 12 NaI ($(D_2O)_{236}(NaI)_{12}$). These molecules were contained in the 14.4 Å × 14.4 Å × 70.0 Å cell. The systems of NaI_3 in D_2O consisted of 114 D_2O and 8 NaI_3 ($(D_2O)_{114}(NaI_3)_8$), 114 D_2O and 4 NaI_3 ($(D_2O)_{114}(NaI_3)_4$), and 114 D_2O and 2 NaI_3 ($(D_2O)_{114}(NaI_3)_2$). These molecules were contained in the 14.4 Å × 14.4 Å × 50.0 Å cell. For the pure D_2O and NaI in D_2O systems, we prepared the 5 random structures for the pure D_2O system and 10 random structures for the NaI in D_2O and NaI_3 in D_2O systems using the Packmol code.[2] After 1 ns equilibration in the force field MD simulation, we performed AIMD simulation for these samples.

For the AIMD simulation, we used the revPBE exchange correlation functional[3] together with the Grimme's D3(0) van der Waals correction.[4] The AIMD simulations employed the QUICKSTEP method[5] implemented in the CP2K code. For the Gaussian part of the basis set, we used the MOLOPT shorter range double zeta basis set,[6] while for the plane wave part we set the plane wave density cutoff to 320 Ry. The norm-conserving Goedecker-Teter-Hutter pseudopotentials[7] for the revPBE functional were generated. Note that, to avoid the nonlinear core valence electron problem,[8] we treated the Na 2s and 2p electrons as valence electrons.[9] We performed the AIMD simulation in the NPT ensemble, where the target temperature was set to 300 K with canonical sampling through a velocity rescaling thermostat.[10] The time step for integrating the equations of motions was set to 0.5 fs. We equilibrated the sample for > 20 ps and then we obtained the ~150 ps AIMD trajectory for each sample, which were used for the analyses.

1.2. Definition of the free O-D groups for NaI and NaI_3 samples

To identify the free O-D group behavior, we used the free O-D (O-H) group definition.[11] In this definition, an O-D group of the interfacial D_2O molecule in which a D atom does not find a hydrogen bond acceptor is categorized into the free O-D group. First, the interfacial D_2O molecules

were selected in the region of $|z\text{-}z_{gd}| < 3.11$ Å, where z and z_{gd} represent the z-coordinate of the oxygen atom of D_2O molecule and the position of the Gibbs dividing surface, respectively. Second, the hydrogen bond formation was judged through all the possible water-water dimer conformations and the water-I^- conformations. For the water-water conformations, an hydrogen bond is formed, when the bond of O…O being less than 3.5 Å and angle of O…O-H being less than 50°.[11] For the water-I^- conformations, we assumed that an hydrogen bond is formed, when the bond of O… I^- being less than 4.2 Å and angle of I^-…O-H being less than 50°. We chose 4.2 Å, because the difference in the first peak positions of the O…O and O…I^- radial distribution functions is 0.7 Å and thus we set the O…I^- distance criterium 0.7 Å larger than the O…O distance criterium.

1.3 Surface density of the free O-D groups

In the main text, we have shown the population of the water molecules with free O-D. The definition of the free O-H group for is given in the main text. We picked up the water molecules in the interfacial region of $|z\text{-} z_{gd}| < 3.11$ Å. For these molecules, we counted the number of water molecules with free O-H groups.

1.4 Determination of bulk and interfacial concentrations of I^- and I_3^-

To calculate the bulk and interfacial concentrations of I^- and I_3^- ions in the simulation condition, we computed the density profile for water, I^- and I_3^- ions, and Na^+ ion, which is shown in Fig. S1. Here, we define the zero of the depth is located at the Gibbs dividing surface for water. Assuming the average number density for the water molecules in the bulk region for the neat water sample is 55.5 M, we calibrated the density profiles for other samples. Based on the density profiles in Figs. 2b-d of the main text, we obtained the bulk NaI concentrations of ~2.6 M and ~5.3 M for $(D_2O)_{236}(NaI)_{12}$ and $(D_2O)_{216}(NaI)_{24}$ samples, respectively. For the surface density of I_3^- ions, we calculated the average density from the number of I_3^- ions in the area of 14.4 Å × 14.4 Å.

It is worth mentioning that our density profile data for NaI samples from AIMD did not show significant surface activity of I^- ion: For the $(D_2O)_{236}(NaI)_{12}$ sample, there is no clear peak

feature arising from preferential adsorption of I^- ion at the interface (Fig. 2c). At the bulk concentration of 5.3 M ($(D_2O)_{216}(NaI)_{24}$ sample), the preferential adsorption of I^- ion became prominent from a peak at ~1.8 Å (Fig. 2d), and the peak value is ~6.9 M, which is ~1.3 times higher than the concentration of the bulk region. This trend differs significantly from the previous simulation studies where even at 1-2 M NaI the density profiles of I^- ion showed a clear peak at a distance of ~1 Å[12,13] or ~2.5 Å[14] below the Gibbs dividing surface. Furthermore, the previously reported peak values relative to the bulk concentration are ~1.8[14] or 2.5-3.5[12,13], which is significantly higher than our data. Such quantitative discrepancies between classical MD and AIMD simulations can be attributed to over-simplified form of Lennard-Jones (LJ) 12-6 potential in the classical MD simulation: presumably the short range repulsion term, i.e., the r^{-12}-term in LJ potential overestimate the ion-ion repulstion,[15] leading to the overestimated surface activity of I^- ion. In fact, a density functional theory study also revealed that the above-mentioned empirical polarizable model-based MD may overestimate the surface propensity of I^- ion.[16]

2. Experimental details

2.1. Experimental setups for SFG measurement

Heterodyne-detected sum-frequency generation (HD-SFG) measurements were performed with two setups which have different beam geometry, setup 1 and setup 2.

The setup 1 used a collinear beam geometry using a Ti:Sapphire regenerative amplifier (Spitfire Ace, Spectra-Physics, centered at 800 nm, ~40 fs pulse duration, 5 mJ pulse energy, 1 kHz repetition rate). A part of the output was used to generate a broadband infrared (IR) pulse in an optical parametric amplifier (Light Conversion TOPAS-C) with a silver gallium disulfide ($AgGaS_2$) crystal. The other part of the output was directed through a pulse shaper consisting of a grating-cylindrical mirror system to generate a narrowband visible pulse with a bandwidth of ~10 cm^{-1}. The IR and visible beams were firstly focused onto a 20 μm-thick y-cut quartz plate to generate a local oscillator (LO) signal. Then, these beams were collinearly passed through a 5 mm-thick $SrTiO_3$ plate for the phase modulation and were focused onto the sample surface at angles of incidence of 45°. The SFG signal from the sample interfered with the SFG signal from the LO,

generating the SFG interferogram. The SFG interferogram was dispersed in a spectrometer (Teledyne Princeton Instruments, HRS-300) and detected by a liquid-nitrogen cooled CCD camera (Teledyne Princeton Instruments, PyLoN).

The setup 2 used a non-collinear beam geometry using a Ti:Sapphire regenerative amplifier (Spitfire Ace, Spectra-Physics, centered at 800 nm, ~40 fs pulse duration, 5 mJ pulse energy, 1 kHz repetition rate). A part of the output was used to generate a broadband IR pulse in an optical parametric amplifier (Light Conversion TOPAS-C) with a $AgGaS_2$ crystal. The other part of the output was directed through a pulse shaper consisting of a grating-cylindrical lens system to generate a narrowband visible pulse with a bandwidth of ~10 cm^{-1}. The IR and visible beams were firstly non-collinearly focused onto a 200 nm-thick ZnO on a 1 mm-thick CaF_2 window to generate a LO signal, in a similar manner to Ref.18. These beams including the LO beam were guided via a parabolic mirror, and the LO beam was temporally delayed relative to other beams by inserting a 1.5 mm-thick fused silica plate in its beam path. Then, these beams were re-focused onto the sample surface with another parabolic mirror at angles of incidence of 50°, 61°, and 64° for the IR, LO, and visible beams, respectively. The SFG signal from the sample interfered with the SFG signal from the LO, generating the SFG interferogram. The SFG interferogram was dispersed in a spectrometer (Shamrick 303i, Andor Technology) and detected by a CCD camera (Newton 970, Andor Technology). To accurately obtain the phase in the non-collinear geometry, we controlled the sample height within the accuracy of 1 μm by a height displacement sensor (CL-3000, Keyence).

The complex-valued second-order nonlinear susceptibility ($\chi^{(2)}$) from the samples were obtained via the Fourier analysis of the interferogram and normalization by that from a z-cut quartz crystal. The measurements were performed with *ssp* (denoting *s*-, *s*-, and *p*-polarized SFG, visible, and IR beams, respectively) and *ppp* polarization combinations. After 15 minutes of equilibration of the samples under a nitrogen atmosphere, the HD-SFG measurements were carried out. The exposure time of the measurement was set to three minutes, and the data obtained was averaged over 15 minutes.

2.2 UV-visible absorption measurement

The UV–visible measurement was carried out with a Perkin-Elmer Lambda 900 spectrometer using a quartz cuvette (1 cm path length) at 22 ºC. The instrument was set to have a 4 nm interval to record a spectrum.

3. Data processing and analysis

3.1. Amplitude calibration of the SFG spectra at different polarizations

As briefly described above, the sample HD-SFG spectra at *ssp* and *ppp* polarization combinations were normalized with that for the *z*-cut quartz at *ssp* and *ppp* polarization combinations, respectively. Thus, to quantitatively compare the HD-SFG spectra obtained at *ssp* and *ppp* polarization combinations, one needs to calibrate the amplitude of the normalized spectra, $\chi^{(2)}_{ssp,\mathrm{norm}}$ and $\chi^{(2)}_{ppp,\mathrm{norm}}$:

$$\chi^{(2)}_{ssp,\mathrm{norm}} = -\frac{\chi^{(2)}_{ssp,\mathrm{measured,sample}}}{i\chi^{(2)}_{ssp,\mathrm{measured,zqz}}}, \qquad \text{(S1)}$$

$$\chi^{(2)}_{ppp,\mathrm{norm}} = -\frac{\chi^{(2)}_{ppp,\mathrm{measured,sample}}}{i\chi^{(2)}_{ppp,\mathrm{measured,zqz}}}, \qquad \text{(S2)}$$

where $\chi^{(2)}_{ssp,\mathrm{measured,sample}}$ ($\chi^{(2)}_{ppp,\mathrm{measured,sample}}$) and $\chi^{(2)}_{ssp,\mathrm{measured,zqz}}$ ($\chi^{(2)}_{ppp,\mathrm{measured,zqz}}$) are the measured $\chi^{(2)}$ from the sample and *z*-cut quartz, respectively at *ssp* (*ppp*) polarization combination,

by considering the absolute values of the effective surface non-linear susceptibilities for the *z*-cut quartz at different polarization combinations, i.e., $\chi^{(2)}_{\mathrm{eff},ssp,\mathrm{zqz}}$ and $\chi^{(2)}_{\mathrm{eff},ppp,\mathrm{zqz}}$. The conversion equations are given as

$$\chi^{(2)}_{\mathrm{eff},ssp} = \chi^{(2)}_{ssp,\mathrm{norm}} \frac{r_{q,s}}{r_{sample,s}} \left|\chi^{(2)}_{\mathrm{eff},ssp,\mathrm{zqz}}\right|, \qquad \text{(S3)}$$

$$\chi^{(2)}_{\mathrm{eff},ppp} = \chi^{(2)}_{ppp,\mathrm{norm}} \frac{r_{q,p}}{r_{sample,p}} \left|\chi^{(2)}_{\mathrm{eff},ppp,\mathrm{zqz}}\right|, \qquad \text{(S4)}$$

where $\frac{r_{q,s}}{r_{sample,s}}$ and $\frac{r_{q,p}}{r_{sample,p}}$ show the ratios of reflectivity coefficients between *z*-cut quartz and D_2O/NaI samples for *s*- and *p*-polarized beams at ~660 nm, respectively[19]. These equations allow for comparing the amplitudes of the SFG peaks obtained at different polarization combinations.

For the *z*-cut quartz, the SFG signal is maximized when the *x*-axis of the crystal is parallel to the laser incident plane, where the $\left|\chi^{(2)}_{\mathrm{eff},ssp,\mathrm{zqz}}\right|$ and $\left|\chi^{(2)}_{\mathrm{eff},ppp,\mathrm{zqz}}\right|$ are difined by[20]

$$\left|\chi^{(2)}_{\text{eff},ssp,\text{zqz}}\right| = 2\cos\beta_{\text{IR}}\, L_{yy}(\omega_{\text{SFG}})L_{yy}(\omega_{\text{Vis}})L_{xx}(\omega_{\text{IR}})\chi_q l_c, \tag{S5}$$

$$\left|\chi^{(2)}_{\text{eff},ppp,\text{zqz}}\right| = 2\cos\beta_{\text{SFG}}\cos\beta_{\text{Vis}}\cos\beta_{\text{IR}}\, L_{xx}(\omega_{\text{SFG}})L_{xx}(\omega_{\text{Vis}})L_{xx}(\omega_{\text{IR}})\chi_q l_c, \tag{S6}$$

where β_i and ω_i (i=IR, Vis, SFG) are the incident angle and frequency of the corresponding beam, respectively. L_{jj} (j=x, y, z) is the jj component of Fresnel factors, where the xz-plane forms the incident plane of the beams, and the z-axis is defined as the surface normal. l_c is the SFG coherent length, and l_c was ~39 nm and ~42 nm for the setup 1 and setup 2, respectively. We assumed χ_q is ~8.0×10^{-13} m/V.[20].

The Fresnel factors (L_{jj}) represent coefficients relating the field components in input/output beams to the local field at the interface. For an interface between two continuous media with refractive indexes n_I for medium I and n_{II} for medium II, one has

$$L_{xx} = \frac{2n_I\cos\beta^{II}}{n_I\cos\beta^{II} + n_{II}\cos\beta^{I}}, \tag{S7}$$

$$L_{yy} = \frac{2n_I\cos\beta^{I}}{n_I\cos\beta^{I} + n_{II}\cos\beta^{II}}, \tag{S8}$$

$$L_{zz} = \frac{2n_{II}\cos\beta^{I}}{n_I\cos\beta^{II} + n_{II}\cos\beta^{I}}\left(\frac{n_I}{n'}\right)^2, \tag{S9}$$

where β^I and β^{II} are the beam angles in medium I and medium II, respectively. n' is the interfacial refractive index, which can be calculated via[21]

$$n' = \sqrt{\frac{n_I^2 + n_{II}^2 + 4}{2\left(n_I^{-2} + n_{II}^{-2} + 1\right)}}. \tag{S10}$$

To compute Eqs. (S5) and (S6), we assumed medium I and medium II are air and z-cut quartz, respectively. The refractive index in air is 1, and those of the z-cut quartz are 1.54, 1.54, and 1.48 for SFG (~800 nm), visible (~660 nm), and IR beams (~3700 nm), respectively.[22,23] For convenience the birefringence of z-cut quartz was neglected, and the refractive index of the ordinary wave was used for all polarizations. The refractive index for the IR beam was extrapolated from Ref. 23. Finally, we obtained $\left|\chi^{(2)}_{\text{eff},ssp,\text{zqz}}\right| = 1.85 \times 10^{-20}$ m^2/V and 1.16×10^{-20} m^2/V

and $\left|\chi^{(2)}_{\mathrm{eff},ppp,\mathrm{zqz}}\right| = 1.60 \times\ 10^{-20}$ m^2/V and $0.98 \times\ 10^{-20}$ m^2/V for the setup 1 and setup 2, respectively. With these absolute values, we calibrated the amplitudes in the $\chi^{(2)}_{ssp,\mathrm{norm}}$ and $\chi^{(2)}_{ppp,\mathrm{norm}}$ spectra.

3.2. Fresnel factor correction

Conversion of the $\chi^{(2)}_{\mathrm{eff},ssp}$ and $\chi^{(2)}_{\mathrm{eff},ppp}$ spectra to the $\chi^{(2)}_{yyz}$ and $\chi^{(2)}_{zzz}$ spectra can be made through the Fresnel equations via

$$\chi^{(2)}_{\mathrm{eff},ssp} = L_{yy}(\omega_{\mathrm{SFG}})L_{yy}(\omega_{\mathrm{Vis}})L_{zz}(\omega_{\mathrm{IR}})\sin\beta_{\mathrm{IR}}\chi^{(2)}_{yyz}, \tag{S11}$$

$$\chi^{(2)}_{\mathrm{eff},ppp} \approx -L_{xx}(\omega_{\mathrm{SFG}})L_{xx}(\omega_{\mathrm{Vis}})L_{zz}(\omega_{\mathrm{IR}})\cos\beta_{\mathrm{SFG}}\cos\beta_{\mathrm{Vis}}\sin\beta_{\mathrm{IR}}\chi^{(2)}_{xxz}$$

$$+L_{zz}(\omega_{\mathrm{SFG}})L_{zz}(\omega_{\mathrm{Vis}})L_{zz}(\omega_{\mathrm{IR}})\sin\beta_{\mathrm{SFG}}\sin\beta_{\mathrm{Vis}}\sin\beta_{\mathrm{IR}}\chi^{(2)}_{zzz}. \tag{S12}$$

For evaluating L_{zz} via Eqs. (S9) and (S10), we used the bulk refractive index data of liquid D_2O for medium II.[24] Similarly to our previous work,[25] the refractive indices of NaI solutions for visible and SFG beams are calculated using $n = n_0 + cn_1$, where n_0 is the refractive index of neat D_2O, c in the concentration of NaI, and n_1 is a salt specific correction factor (0.0201).[26] For evaluating $L_{zz}(\omega_{\mathrm{IR}})$, we need the frequency-dependent refractive indices of the NaI-D_2O solution. However, it was not available. Therefore, we used the refractive index which were computed from the refractive index of the NaI-H_2O solution[27] by shifting frequency with a scaling factor of 0.75. For the free O-D groups located in the topmost water layer in NaI samples, we computed n' within the Lorenz model based on the averaged water molecule/Na^+/I^- densities in bulk and at interface at the position of the Gibbs dividing surface defined above, in a similar manner to Ref. 28. The $\mathrm{Im}\chi^{(2)}_{yyz}$ and $\mathrm{Im}\chi^{(2)}_{zzz}$ spectra obtained from the setup 1 after Fresnel factor correction are displayed in Fig. S1.

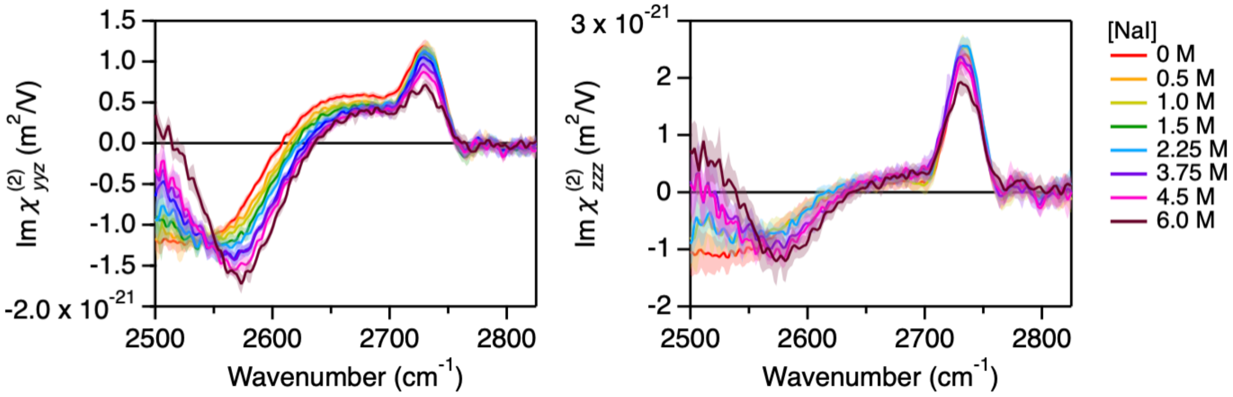

Fig. S1. $\mathrm{Im}\chi^{(2)}_{yyz}$ (a) and $\mathrm{Im}\chi^{(2)}_{zzz}$ (b) spectra after removal of the Fresnel coefficients. The shaded area displays the 95% confidence interval of the multiple measurements.

3.3. Fits of SFG spectra

To extract the amplitude of the free O-D stretch mode peak from the $\mathrm{Im}\chi^{(2)}_{yyz}$ and $\mathrm{Im}\chi^{(2)}_{zzz}$ spectra (displayed in Figs. 1c and 1d of the main text), we fit the spectra by using the Gaussian lineshapes:

$$\mathrm{Im}\chi^{(2)} = \sum_{i=1}^{n} a_i \exp\left(-\frac{(\omega-\omega_i)^2}{2\sigma_i^2}\right), \quad \text{(S13)}$$

where a_i, ω_i, and σ_i are the amplitude, the characteristic frequency, and the band width of the ith O-D stretch mode, respectively. n denotes the number of Gaussians used for the fitting. For the $\mathrm{Im}\chi^{(2)}_{yyz}$ and $\mathrm{Im}\chi^{(2)}_{zzz}$ spectra, we used $n = 3$ for fitting the spectra of the neat D_2O sample and $n = 4$ for fitting the spectra of the NaI samples.

For the neat D_2O sample, we use three Gaussian contributions representing the peak arising from the hydrogen-bonded O-D stretch mode at ~2500-2630 cm^{-1}, the anti-symmetric O-D stretch mode of the water molecules with two hydrogen bond donors at ~2650 cm^{-1} (the shoulder contribution)[29], and the free O-D stretch mode at ~2730 cm^{-1}. As is clear from the spectra shown in Figs. 1c and 1d in the main text, NaI samples, in particular with a concentration of more than 3.0 M, show complicated spectral features at ~2500-2600 cm^{-1}, i.e., the positive and negative

responses. Thus, to take the positive contribution into account, we included another Gaussian contribution, i.e., four Gaussian contributions for the NaI samples, and performed the fitting. In a similar manner, we performed the fitting for the NaI samples in the presence of I_2 /$Na_2S_2O_3$. For these samples, we used the same fitting parameters for the hydrogen-bonded O-D stretch mode contributions at ~2500-2630 cm^{-1}. The obtained fits and fitting parameters for the dataset from the setup 1 are summarized in Fig. S2 and Tables S1-2, respectively. The fits and fitting parameters for the setup 2 are summarized in Fig. S3 and Table S3, respectively.

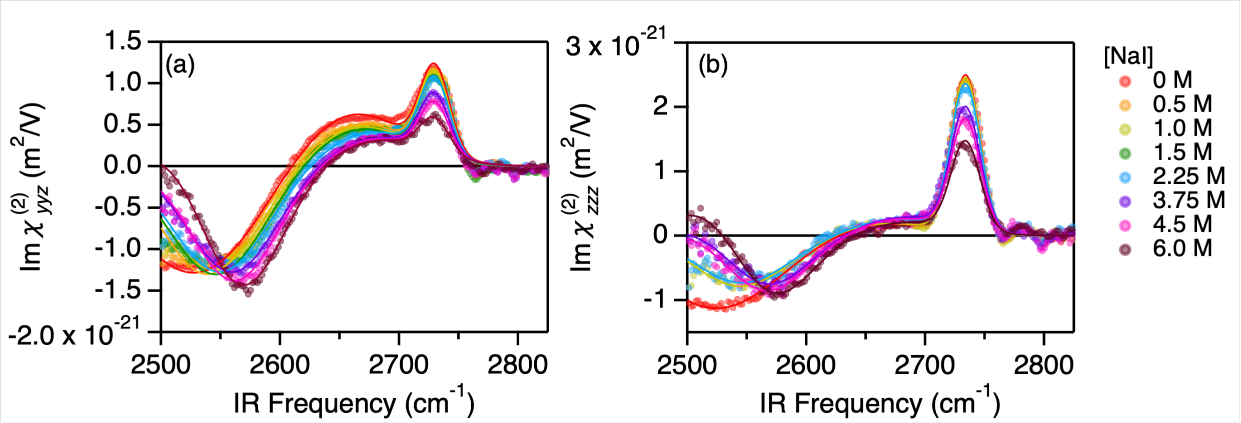

Fig. S2. $\mathrm{Im}\chi^{(2)}_{yyz}$ (a) and $\mathrm{Im}\chi^{(2)}_{zzz}$ (b) spectra for the entire fitting region for neat D_2O and NaI samples. The circles and solid lines represent the experimental data and the fits, respectively.

Table S1. The fitting parameters used for describing the $\mathrm{Im}\chi^{(2)}_{yyz}$ spectra for the N_2 gas-neat D_2O and N_2 gas-NaI aqueous solution interfaces.

[NaI]	0 M	0.5 M	1.0 M	1.5 M	2.25 M	3.75 M	4.5 M	6.0 M	1.5 M + 1 μM I_3^-	1.5 M + 1 μM I_3^- + 100 mM $Na_2S_2O_3$
a_1 ($\times 10^{-21}$ m^2/V)		-0.90 ± 0.04	-0.68 ± 0.03	-0.60 ± 0.03	-0.49 ± 0.04	-0.01 ± 0.03	0.11 ± 0.03	0.75 ± 0.03	-0.60 ± 0.03	-0.60 ± 0.03
ω_1 (cm^{-1})		2530	2530	2530	2530	2530	2530	2530	2530	2530
σ_1 (cm^{-1})		34 ± 1	34 ± 1	34 ± 1	34 ± 1	34 ± 1	34 ± 1	34 ± 1	34 ± 1	34 ± 1
a_2 ($\times 10^{-21}$ m^2/V)	-1.28 ± 0.01	-0.51 ± 0.03	-0.72 ± 0.03	-0.87 ± 0.03	-0.90 ± 0.03	-1.27 ± 0.03	-1.46 ± 0.03	-1.88 ± 0.03	-0.87 ± 0.03	-0.87 ± 0.03
ω_2 (cm^{-1})	2529 ± 2	2560	2560	2560	2560	2560	2560	2560	2560	2560
σ_2 (cm^{-1})	53 ± 2	36 ± 1	36 ± 1	36 ± 1	36 ± 1	36 ± 1	36 ± 1	36 ± 1	36 ± 1	36 ± 1
a_3 ($\times 10^{-21}$ m^2/V)	0.68 ± 0.02	0.52 ± 0.02	0.50 ± 0.01	0.48 ± 0.02	0.42 ± 0.02	0.35 ± 0.02	0.33 ± 0.02	0.32 ± 0.02	0.32 ± 0.02	0.44 ± 0.04
ω_3 (cm^{-1})	2659 ± 3	2669 ± 4	2669 ± 2	2669 ± 2	2678 ± 2	2684 ± 2	2682 ± 2	2685 ± 2	2671 ± 2	2669 ± 2
σ_3 (cm^{-1})	47 ± 2	41 ± 2	41 ± 2	41 ± 2	41 ± 2	41 ± 2	41 ± 2	41 ± 2	41 ± 2	41 ± 2
a_{free} ($\times 10^{-21}$ m^2/V)	1.02 ± 0.03	1.01 ± 0.03	1.02 ± 0.03	0.98 ± 0.03	0.90 ± 0.04	0.70 ± 0.03	0.61 ± 0.03	0.41 ± 0.03	0.75 ± 0.04	0.99 ± 0.07
ω_{free} (cm^{-1})	2730 ± 1	2730 ± 1	2730 ± 1	2730 ± 1	2730 ± 1	2730 ± 1	2730 ± 1	2730 ± 1	2730 ± 1	2730 ± 1
σ_{free} (cm^{-1})	12 ± 1	12 ± 1	12 ± 1	12 ± 1	12 ± 1	12 ± 1	12 ± 1	12 ± 1	12 ± 1	12 ± 1

Table S2. The fitting parameters used for describing the $\mathrm{Im}\chi^{(2)}_{zzz}$ spectra for the N_2 gas-neat D_2O and N_2 gas-NaI aqueous solution interfaces.

[NaI]	0 M	1.0 M	2.25 M	3.75 M	4.5 M	6.0 M
a_1 ($\times 10^{-21}$ m^2/V)		-0.41 ± 0.03	-0.36 ± 0.04	0.40 ± 0.03	0.34 ± 0.03	1.01 ± 0.03
ω_1 (cm^{-1})		2530	2530	2530	2530	2530
σ_1 (cm^{-1})		34 ± 1	34 ± 1	34 ± 1	34 ± 1	34 ± 1
a_2 ($\times 10^{-21}$ m^2/V)	-1.13 ± 0.01	-0.48 ± 0.03	-0.44 ± 0.03	-0.99 ± 0.03	-1.07 ± 0.03	-1.43 ± 0.04
ω_2 (cm^{-1})	2525 ± 1	2560	2560	2560	2560	2560
σ_2 (cm^{-1})	53 ± 2	36 ± 1	36 ± 1	36 ± 1	36 ± 1	36 ± 1
a_3 ($\times 10^{-21}$ m^2/V)	0.21 ± 0.01	0.20 ± 0.01	0.26 ± 0.02	0.29 ± 0.02	0.27 ± 0.02	0.21 ± 0.00
ω_3 (cm^{-1})	2659 ± 3	2669 ± 2	2678 ± 2	2684 ± 2	2682 ± 2	2685 ± 2
σ_3 (cm^{-1})	47 ± 2	41 ± 2	41 ± 2	41 ± 2	41 ± 2	41 ± 2
a_{free} ($\times 10^{-21}$ m^2/V)	2.44 ± 0.02	2.50 ± 0.03	2.26 ± 0.03	1.87 ± 0.03	1.70 ± 0.03	1.37 ± 0.03
ω_{free} (cm^{-1})	2734 ± 1	2734 ± 1	2734 ± 1	2734 ± 1	2734 ± 1	2734 ± 1
σ_{free} (cm^{-1})	12 ± 1	12 ± 1	12 ± 1	12 ± 1	12 ± 1	12 ± 1

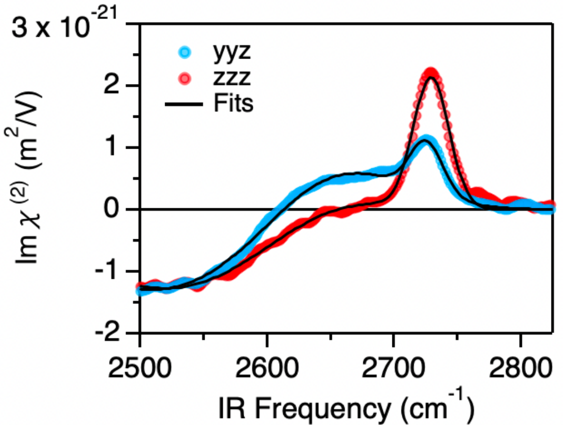

Fig. S3. $\mathrm{Im}\chi^{(2)}_{yyz}$ and $\mathrm{Im}\chi^{(2)}_{zzz}$ spectra obtained from the setup 2 for the entire fitting region for neat D_2O. The circles and solid lines represent the experimental data and the fits, respectively.

Table S3. The fitting parameters used for describing the $\mathrm{Im}\chi^{(2)}_{yyz}$ and $\mathrm{Im}\chi^{(2)}_{zzz}$ spectra obtained from the setup 2 for the N_2 gas-neat D_2O interface.

Polarization	yyz	zzz
a_1 ($\times10^{-21}$ m^2/V)		
ω_1 (cm^{-1})		
σ_1 (cm^{-1})		
a_2 ($\times10^{-21}$ m^2/V)	-1.34 ± 0.04	-1.26 ± 0.03
ω_2 (cm^{-1})	2520 ± 7	2522 ± 7
σ_2 (cm^{-1})	91 ± 11	91 ± 11
a_3 ($\times10^{-21}$ m^2/V)	0.99 ± 0.02	0.44 ± 0.02
ω_3 (cm^{-1})	2651 ± 3	2655 ± 4
σ_3 (cm^{-1})	55 ± 5	55 ± 5
a_{free} ($\times10^{-21}$ m^2/V)	0.83 ± 0.07	2.09 ± 0.03
ω_{free} (cm^{-1})	2726 ± 1	2730 ± 1
σ_{free} (cm^{-1})	13 ± 1	13 ± 1

3.4. Orientational and number density analysis

With the obtained peak amplitude ratio (a_{yyz}/a_{zzz}), we evaluated the angle of the free O-D group of heavy water with respect to the surface normal. Details of the orientational analysis are reported in our previous study.[30] Briefly, A_{yyz} and A_{zzz} (spectral areas of the free O-D group) components are respectively represented via equations[21]:

$$A_{yyz} \approx \frac{1}{2} N_s \alpha [(1+r)\langle \cos\theta \rangle - (1-r)\langle \cos^3\theta \rangle], \quad \text{(S14)}$$

$$A_{zzz} \approx N_s \alpha [r\langle \cos\theta \rangle + (1-r)\langle \cos^3\theta \rangle] \quad \text{(S15)}$$

in the slow motion limit where the decay of the orientational memory of the free O-D group is much slower than vibrational relaxation.[31] N_s, α, and r denote the number density, hyperpolarizability, and depolarization ratio of a vibration at interface, respectively. We used $r = 0.15$ for the free O-D stretch mode.[30] By taking the ratio of A_{yyz}/A_{zzz}, Eqs. (S14) and (S15) are recast as;

$$\frac{A_{yyz}}{A_{zzz}} \approx \frac{(1+r)\langle \cos\theta \rangle - (1-r)\langle \cos^3\theta \rangle}{2r\langle \cos\theta \rangle + 2(1-r)\langle \cos^3\theta \rangle}, \quad \text{(S16)}$$

where $\langle B \rangle = \int_0^\pi B f(\theta) \sin\theta \, d\theta$ and $f(\theta)$ is the orientational distribution function of the free O-H groups. According to Ref. 30, we used the form of $f(\theta) = N_E \exp(-\theta/\theta_E)$, where N_E is the normalization factor. The right side of Eq. (S16) against the average orientational angle of the free O-D groups, $\langle \theta \rangle$, is displayed as the black line in Fig. S4 (a). Once we obtain $\langle \cos\theta \rangle$ and $\langle \cos^3\theta \rangle$ values by knowing $f(\theta)$, one can obtain N_s from Eq. (S14). Note that here we assumed that α is unchanged due to the addition of NaI. The obtained a_{yyz}/a_{zzz} was used for this analysis because the amplitude ratio is equivalent to A_{yyz}/A_{zzz} with the same bandwidth of the peaks. The average orientational angles of the free O-D groups in the absence and presence of NaI and the corresponding $\langle \cos\theta \rangle$ and $\langle \cos^3\theta \rangle$ and the number density N_s are summarized in Fig. D4 (b) and Table S4 for the setup 1. For the dataset obtained from the setup 2, we obtained the A_{yyz}/A_{zzz} ratio of 0.40 ± 0.03 for the neat D_2O sample, which is in good agreement with the ratio of 0.42 ± 0.01 obtained from the setup 1. This suggests robustness of our measurements.

Here, we would like to emphasize that the changes in the peak amplitude in Fig. 1e are dominantly attributed to the variations of the number density of the free-OD chromophores at the interface. Upon changing [NaI] from 1.0 M to 6.0 M, the a_{yyz} was reduced by ~60%. This reduction is the result of the competition of the orientational contribution and the density contribution; the orientational term $[(1+r)\langle\cos\theta\rangle-(1-r)\langle\cos^3\theta\rangle]$ in Eq. (S14) increased by ~40% while the number density decreased by ~70%. As such, the changes in the number density of the free-OD chromophores overwhelms the variations of the orientational parameters. Through the careful separation of these contributions, one can access the change in N_s upon the variation of [NaI]. The free O-H group covers the 25% of the water surface[32] and such a free O-D group is reduced by 70% due to the presence of triiodide, the number of the triiodide at the interface can be unambiguously estimated.

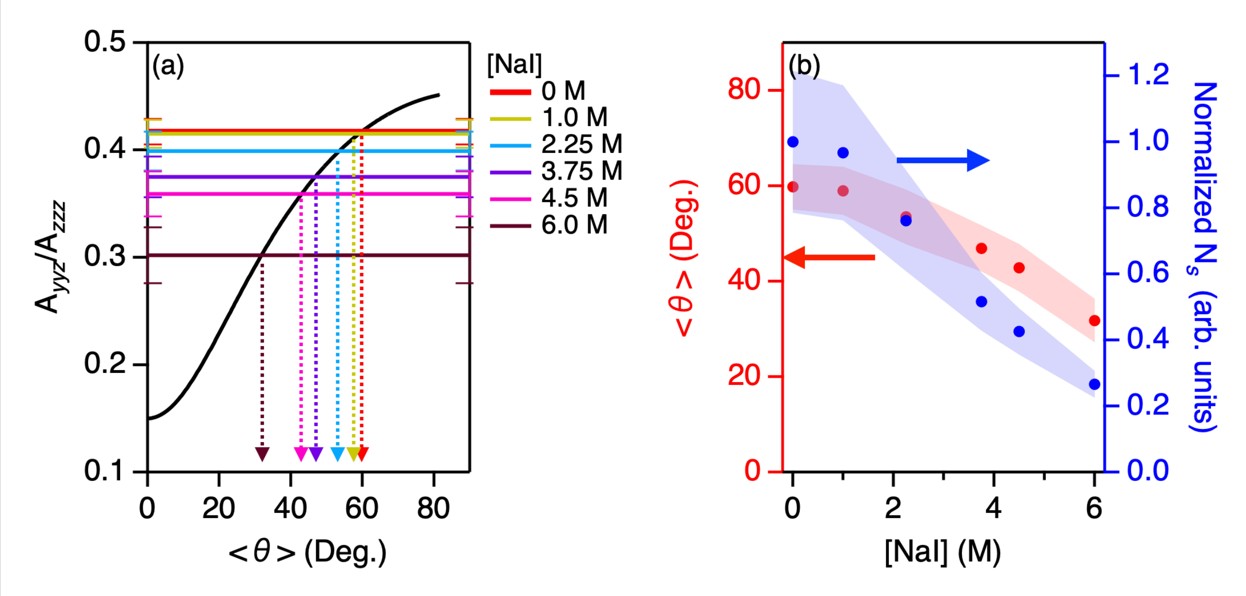

Fig. S4. (a) The ratio of A_{yyz}/A_{zzz} as a function of the average orientational angle of the free O-D groups. The colored lines indicate the obtained A_{yyz}/A_{zzz} values for the samples in the presence and absence of NaI in the solution. The crossing points between the black and colored lines are the corresponding $\langle\theta\rangle$ for the samples. (b) The average angle of the free O-D group $\langle\theta\rangle$ and the normalized number of the free O-D chromophores N_s as a function of the NaI concentrations. N_s were normalized with its maximum. The shade represents the error bars obtained from the fits.

Table S4. Thermal averaged values and the surface density for the free O-D groups at the air-water interface in the presence and absence of NaI. The surface density N_s was normalized to the maximum value at 1.0 M.

[NaI]	$\langle\cos\theta\rangle$	$\langle\cos^3\theta\rangle$	$\langle\theta\rangle$ (deg.)	N_s
0 M	0.437 ± 0.073	0.288 ± 0.054	59.8 ± 4.8	1.0 ± 0.21
1.0 M	0.444 ± 0.071	0.293 ± 0.052	59.0 ± 5.1	0.97 ± 0.21
2.25 M	0.519 ± 0.079	0.351 ± 0.062	53.5 ± 5.8	0.76 ± 0.15
3.75 M	0.611 ± 0.064	0.427 ± 0.056	46.9 ± 4.9	0.51 ± 0.09
4.5 M	0.662 ± 0.064	0.474 ± 0.061	42.8 ± 5.0	0.43 ± 0.07
6.0 M	0.796 ± 0.051	0.620 ± 0.064	31.8 ± 4.6	0.27 ± 0.04

4. Surface pressure of NaCl and NaI aqueous solutions.

The surface pressure data is a well-studied indicator of the surface-activity of ions. We examined the reported surface pressure data of NaCl and NaI aqueous solutions with various salt concentrations (Fig. S5).[33,34] Note that regarding NaBr, because two references show a different behavior, we do not refer the surface pressure data here. While NaCl data shows a linear modulation of the surface pressure, NaI data exhibits a rather nonlinear behavior in a similar manner to the variation of the free O-D peak amplitude (a_{yyz}) against [NaI] shown in Fig. 1(e) in the main text. This difference further illustrates the unique surface structure of NaI solutions.

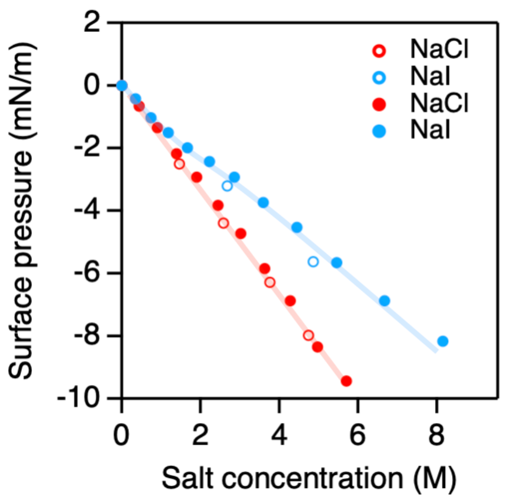

Fig. S5. Surface pressure data of NaCl and NaI aqueous solutions as a function of salt concentrations at 25 °C. The data of the open and closed circles are obtained from Ref. [33] and Ref. [34], respectively. The solid red and blue lines indicate the guide for the eyes.

5. The variation of the free O-H amplitude at NaBr aqueous solution interface

At high salt concentrations, the Na^+ and I^- may form ion-pairing,[35] altering the surface activity of I^-, thereby possibly reducing the *Ns* significantly as displayed in Fig. 2a in the main text. To testify this effect, we measured the N_2 gas-NaBr solution interface as NaBr is chemically stable and likely forms the ion-paring. Here, we used H_2O instead of D_2O and we believe that this change does not affect the trend of ion-paring as the past studies demonstrate the similarity of H_2O and D_2O interface.[36–38] The HD-SFG data are shown in Fig. S6(a). The HD-SFG data for NaBr/H_2O solutions commonly display the 3630 cm^{-1} shoulder peak and the 3700 cm^{-1} peak. Upon changing the bulk concentrations of NaBr, i.e., [NaBr], from 0 M to 4.0 M, the free O-H feature at 3700 cm^{-1} of interfacial H_2O molecule decreased gradually. This is in contrast with the drastic change in the peak amplitude of the free O-D group in the NaI solution, as is seen in Fig. S6(b). While the free O-H peak amplitude for the NaBr samples varied linearly, the free O-D peak amplitude of the NaI samples exhibited a nonlinear trend, as is discussed in Fig. 1e (a_{yyz} vs [NaI]) in the main text. Apparently, NaI samples showed the different trend of the variation of the topmost water layer's structure. These corroborate the scenario of the formation of the triiodide rather than the ion-pairing effect. Note that the data of NaBr samples were obtained from Ref. 39.

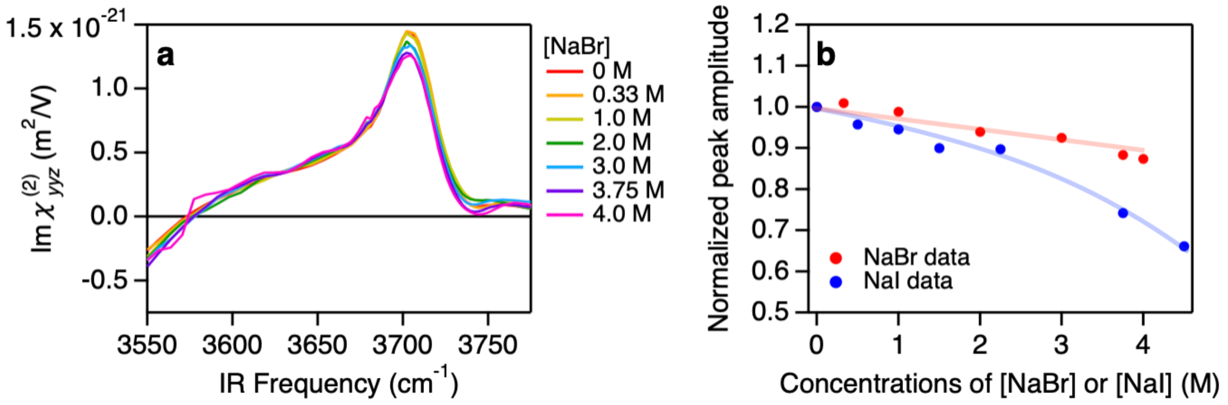

Fig. S6. (a) $\mathrm{Im}\chi^{(2)}_{yyz}$ spectra obtained from the N_2 gas-NaBr solution interface after removal of the Fresnel coefficients. (b) Variations of the 3700 cm^{-1} peak amplitude for NaBr samples and the 2730 cm^{-1} peak amplitude against the bulk concentrations of [NaBr] and [NaI], respectively. The peak amplitudes were normalized with the corresponding amplitudes of [NaBr] = 0 M and [NaI] = 0 M data for clarity. The solid red and blue lines indicate the guides for the eyes.

6. Comparison of the neat D_2O and $NaIO_3$ samples

To confirm that IO_3^- ion does not affect the topmost interfacial water's structure, we measured $\mathrm{Im}\chi^{(2)}_{yyz}$ spectra of $NaIO_3$ sample with its concentration of 0.3 M and found that the spectral shape is indistinguishable with that for the neat D_2O in the free O-D stretch mode region (Fig. S7). This observation is consistent with the previously proposed less surface active nature of IO_3^- ion.[40,41]

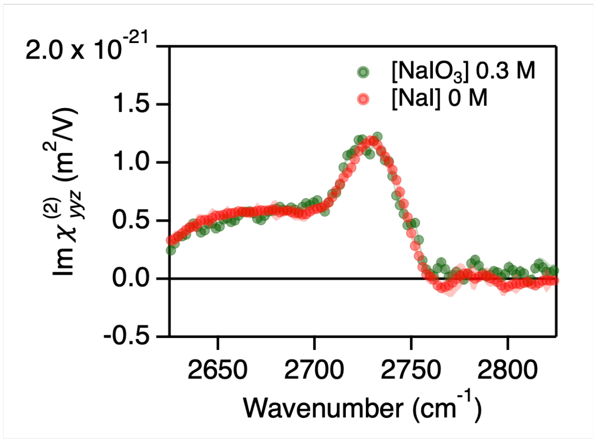

Fig. S7. Comparison of the $\mathrm{Im}\chi^{(2)}_{yyz}$ spectra for the neat D_2O and $NaIO_3$ samples.

7. Determination of bulk I_3^- concentration based on the UV-visible spectra

To measure the I_3^- concentration in the bulk solution upon increasing the [NaI], we measured UV-visible spectra of the NaI-H_2O solutions with various NaI concentrations (Fig. 3e in the main text). The presence of I_3^- species gives a ~350 nm feature in the ultraviolet UV-visible spectrum, allowing us to identify the I_3^- concentration from the 350 nm spectral intensity. For the 5 M and 6 M NaI samples, we clearly saw a subtle 350 nm feature in the UV-visible spectra (Figure 3e in the main text). From the fits of the intensity of the spectra with a gaussian function located at ~360 nm as well as the absorption coefficient of the I_3^- species of 2.76×10^4 $M^{-1}cm^{-1}$,[42] we estimated the bulk concentration of the I_3^- species to ~ 47 nM and ~ 54 nM for the 5 M and 6 M NaI solutions, respectively.

8. Energy difference of I_3^- molecule at the water-air interface and in the bulk

We performed the AIMD simulations for the system composed of 114 D_2O molecule and an I_3^- ion. For initial configurations of the samples, we set three independent samples for the I_3^- ion at the water-air interface as well as three independent samples in the bulk. The simulation condition for the AIMD simulations was the same as that written in Sec. 1.1, except the basis set for the D_2O

molecule; we used the DZVP basis set instead of the MOLOPT basis set, to accelerate the AIMD simulations. We ran the 20 ps simulations following the 11.5 ps simulations for equilibrating the systems.

The energy difference obtained from this simulation was 19.1 kJ/mol, indicating that the population at interface is 3×10^8 times larger at the water-air interface than in the bulk. Based on this data together with the UV-vis estimation of the bulk the I_3^- species, we obtained the surface area density of 2.43 nm^{-2} and 2.80 nm^{-2} for 5 M NaI and 6 M NaI solutions, respectively, where we assumed that the thickness of the water-air interface is represented by the "10-90" thickness[43] and thus is given by 2.86 Å[44].

9. Photosensitivity of NaI Sample under SFG Measurements

To exclude the possibility that the generation of I_3^- is induced by the photochemical reaction due to laser irradiation during the SFG measurement, we checked the time evolution of the SFG signal at the air-NaI solution with its concentration of 3.75 M. We chose 3.75 M sample, because the SFG spectrum for the 3.75 M NaI solution sample signified the presence of I_3^- ions, as is discussed in the main text (Fig. 1a). To do so, we firstly purged N_2 into the system for ~30 min. After that, we started to irradiate the sample with visible and IR beams and monitor the change of the free O-D stretch peak. If an I_3^- species is being generated in time due to the photo-irradiation, the $\mathrm{Im}\chi^{(2)}_{\mathrm{eff},ssp}$ free O-D stretch feature will change over the time. The obtained data (Fig. S8(a)), however, showed that the $\mathrm{Im}\chi^{(2)}_{\mathrm{eff},ssp}$ spectra obtained at different time are indistuinguishable, manifesting that the spectral feature in Figs. 1a-d in the main text was not caused by the photo-irradiation of SFG measurement.

Furthermore, to check whether the laser irradiation during SFG measurement affects the generation of I_3^- in the bulk, we compared the UV-visible spectra of the NaI sample with its concentration of 3.75 M with and without visible and IR laser irradiation. These data are also displayed in Fig. S8(b). We used the same visible laser power as the one used in the SFG measurement. The observation that both UV-visible spectra did not show the 350 nm peak strongly suggests that the laser-irradiation in the nitrogen condition did not generate I_3^- species in bulk.

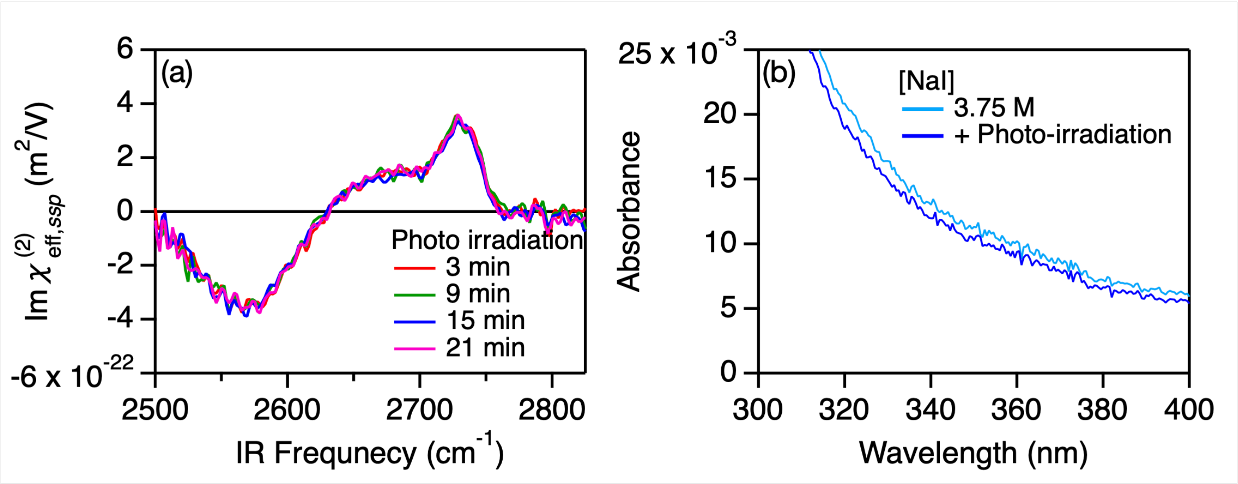

Fig. S8. (a) Time evolution of $\mathrm{Im}\chi^{(2)}_{\mathrm{eff},ssp}$ spectra for the N_2 gas-D_2O interface with [NaI] = 3.75 M. The legend indicates the elapsed time after starting the SFG measurement. (b) Comparison of the UV-visible spectra of NaI solution samples with [NaI] = 3.75 M with and without visible and IR laser irradiation for 1 hour.

SUPPORTING REFERENCES

(1) Hutter, J.; Iannuzzi, M.; Schiffmann, F.; VandeVondele, J. CP2K: Atomistic Simulations of Condensed Matter Systems. *Wiley Interdiscip. Rev. Comput. Mol. Sci.* **2014**, *4* (1), 15–25. https://doi.org/10.1002/wcms.1159.

(2) Martínez, L.; Andrade, R.; Birgin, E. G.; Martínez, J. M. PACKMOL : A Package for Building Initial Configurations for Molecular Dynamics Simulations. *J. Comput. Chem.* **2009**, *30* (13), 2157–2164. https://doi.org/10.1002/jcc.21224.

(3) Ernzerhof, M.; Perdew, J. P. Generalized Gradient Approximation to the Angle- and System-Averaged Exchange Hole. *J. Chem. Phys.* **1998**, *109* (9), 3313–3320.

https://doi.org/10.1063/1.476928.

(4) Grimme, S.; Antony, J.; Ehrlich, S.; Krieg, H. A Consistent and Accurate Ab Initio Parametrization of Density Functional Dispersion Correction (DFT-D) for the 94 Elements H-Pu. *J. Chem. Phys.* **2010**, *132* (15), 154104. https://doi.org/10.1063/1.3382344.

(5) Vandevondele, J.; Krack, M.; Mohamed, F.; Parrinello, M.; Chassaing, T.; Hutter, J. Quickstep: Fast and Accurate Density Functional Calculations Using a Mixed Gaussian and Plane Waves Approach. *Comput. Phys. Commun.* **2005**, *167* (2), 103–128. https://doi.org/10.1016/j.cpc.2004.12.014.

(6) VandeVondele, J.; Hutter, J. Gaussian Basis Sets for Accurate Calculations on Molecular Systems in Gas and Condensed Phases. *J. Chem. Phys.* **2007**, *127* (11), 114105. https://doi.org/10.1063/1.2770708.

(7) Goedecker, S.; Teter, M.; Hutter, J. Separable Dual-Space Gaussian Pseudopotentials. *Phys. Rev. B* **1996**, *54* (3), 1703–1710. https://doi.org/10.1103/PhysRevB.54.1703.

(8) Louie, S. G.; Froyen, S.; Cohen, M. L. Nonlinear Ionic Pseudopotentials in Spin-Density-Functional Calculations. *Phys. Rev. B* **1982**, *26* (4), 1738–1742.

(9) Liu, L. M.; Krack, M.; Michaelides, A. Interfacial Water: A First Principles Molecular Dynamics Study of a Nanoscale Water Film on Salt. *J. Chem. Phys.* **2009**, *130* (23). https://doi.org/10.1063/1.3152845.

(10) Bussi, G.; Donadio, D.; Parrinello, M. Canonical Sampling through Velocity Rescaling. *J. Chem. Phys.* **2007**, *126*, 014101. https://doi.org/10.1063/1.2408420.

(11) Tang, F.; Ohto, T.; Hasegawa, T.; Xie, W. J.; Xu, L.; Bonn, M.; Nagata, Y. Definition of Free O–H Groups of Water at the Air–Water Interface. *J. Chem. Theory Comput.* **2018**, *14* (1), 357–364. https://doi.org/10.1021/acs.jctc.7b00566.

(12) Brown, E. C.; Mucha, M.; Jungwirth, P.; Tobias, D. J. Structure and Vibrational Spectroscopy of Salt Water/Air Interfaces: Predictions from Classical Molecular Dynamics Simulations. *J. Phys. Chem. B* **2005**, *109* (16), 7934–7940. https://doi.org/10.1021/jp0450336.

(13) Jungwirth, P.; Tobias, D. J. Molecular Structure of Salt Solutions: A New View of the Interface with Implications for Heterogeneous Atmospheric Chemistry. *J. Phys. Chem. B* **2001**, *105* (43), 10468–10472. https://doi.org/10.1021/jp012750g.

(14) Ishiyama, T.; Morita, A. Molecular Dynamics Study of Gas−Liquid Aqueous Sodium Halide Interfaces. I. Flexible and Polarizable Molecular Modeling and Interfacial Properties. *J. Phys. Chem. C* **2007**, *111* (2), 721–737. https://doi.org/10.1021/jp065191s.

(15) Bernhardt, M. P.; Nagata, Y.; van der Vegt, N. F. A. Where Lennard-Jones Potentials Fail: Iterative Optimization of Ion–Water Pair Potentials Based on Ab Initio Molecular Dynamics Data. *J. Phys. Chem. Lett.* **2022**, *13* (16), 3712–3717. https://doi.org/10.1021/acs.jpclett.2c00121.

(16) Baer, M. D.; Mundy, C. J. Toward an Understanding of the Specific Ion Effect Using Density Functional Theory. *J. Phys. Chem. Lett.* **2011**, *2* (9), 1088–1093. https://doi.org/10.1021/jz200333b.

(17) Palmer, D. A.; Ramette, R. W.; Mesmer, R. E. Triiodide Ion Formation Equilibrium and

Activity Coefficients in Aqueous Solution. *J. Solution Chem.* **1984**, *13* (9), 673–683. https://doi.org/10.1007/BF00650374.

(18) Vanselous, H.; Petersen, P. B. Extending the Capabilities of Heterodyne-Detected Sum-Frequency Generation Spectroscopy: Probing Any Interface in Any Polarization Combination. *J. Phys. Chem. C* **2016**, *120* (15), 8175–8184. https://doi.org/10.1021/acs.jpcc.6b01252.

(19) Nihonyanagi, S.; Mondal, J. A.; Yamaguchi, S.; Tahara, T. Structure and Dynamics of Interfacial Water Studied by Heterodyne-Detected Vibrational Sum-Frequency Generation. *Annu. Rev. Phys. Chem.* **2013**, *64* (1), 579–603. https://doi.org/10.1146/annurev-physchem-040412-110138.

(20) Wei, X.; Hong, S. C.; Lvovsky, A. I.; Held, H.; Shen, Y. R. Evaluation of Surface vs Bulk Contributions in Sum-Frequency Vibrational Spectroscopy Using Reflection and Transmission Geometries. *J. Phys. Chem. B* **2000**, *104* (14), 3349–3354. https://doi.org/10.1021/jp9933929.

(21) Zhuang, X.; Miranda, P. B.; Kim, D.; Shen, Y. R. Mapping Molecular Orientation and Conformation at Interfaces by Surface Nonlinear Optics. *Phys. Rev. B* **1999**, *59* (19), 12632–12640. https://doi.org/10.1103/PhysRevB.59.12632.

(22) Ghosh, G. Dispersion-Equation Coefficients for the Refractive Index and Birefringence of Calcite and Quartz Crystals. *Opt. Commun.* **1999**, *163* (1–3), 95–102. https://doi.org/10.1016/S0030-4018(99)00091-7.

(23) Radhakrishnan, T. The Dispersion, Briefringence and Optical Activity of Quartz. *Proc.*

Indian Acad. Sci. - Sect. A **1947**, *25* (3), 260–265. https://doi.org/10.1007/BF03171408.

(24) Bertie, J. E.; Ahmed, M. K.; Eysel, H. H. Infrared Intensities of Liquids. 5. Optical and Dielectric Constants, Integrated Intensities, and Dipole Moment Derivatives of H2O and D2O at 22°C. *J. Phys. Chem.* **1989**, *93* (6), 2210–2218. https://doi.org/10.1021/j100343a008.

(25) Piatkowski, L.; Zhang, Z.; Backus, E. H. G.; Bakker, H. J.; Bonn, M. Extreme Surface Propensity of Halide Ions in Water. *Nat. Commun.* **2014**, *5* (1), 4083. https://doi.org/10.1038/ncomms5083.

(26) Max, J. J.; Chapados, C. IR Spectroscopy of Aqueous Alkali Halide Solutions: Pure Salt-Solvated Water Spectra and Hydration Numbers. *J. Chem. Phys.* **2001**, *115* (6), 2664–2675. https://doi.org/10.1063/1.1337047.

(27) Rhine, P.; Williams, D.; Hale, G. M.; Querry, M. R. Infrared Optical Constants of Aqueous Solutions of Electrolytes. Acids and Bases. *J. Phys. Chem.* **1974**, *78* (14), 1405–1410. https://doi.org/10.1021/j100607a014.

(28) Shiratori, K.; Morita, A. Molecular Theory on Dielectric Constant at Interfaces: A Molecular Dynamics Study of the Water/Vapor Interface. *J. Chem. Phys.* **2011**, *134* (23), 234705. https://doi.org/10.1063/1.3598484.

(29) Stiopkin, I. V.; Weeraman, C.; Pieniazek, P. A.; Shalhout, F. Y.; Skinner, J. L.; Benderskii, A. V. Hydrogen Bonding at the Water Surface Revealed by Isotopic Dilution Spectroscopy. *Nature* **2011**, *474* (7350), 192–195. https://doi.org/10.1038/nature10173.

(30) Sun, S.; Tang, F.; Imoto, S.; Moberg, D. R.; Ohto, T.; Paesani, F.; Bonn, M.; Backus, E.

H. G.; Nagata, Y. Orientational Distribution of Free O-H Groups of Interfacial Water Is Exponential. *Phys. Rev. Lett.* **2018**, *121* (24), 246101. https://doi.org/10.1103/PhysRevLett.121.246101.

(31) Wei, X.; Shen, Y. R. Motional Effect in Surface Sum-Frequency Vibrational Spectroscopy. *Phys. Rev. Lett.* **2001**, *86* (21), 4799–4802. https://doi.org/10.1103/PhysRevLett.86.4799.

(32) Du, Q.; Superfine, R.; Freysz, E.; Shen, Y. R. Vibrational Spectroscopy of Water at the Vapor/Water Interface. *Phys. Rev. Lett.* **1993**, *70* (15), 2313–2316. https://doi.org/10.1103/PhysRevLett.70.2313.

(33) Okur, H. I.; Chen, Y.; Wilkins, D. M.; Roke, S. The Jones-Ray Effect Reinterpreted: Surface Tension Minima of Low Ionic Strength Electrolyte Solutions Are Caused by Electric Field Induced Water-Water Correlations. *Chem. Phys. Lett.* **2017**, *684*, 433–442. https://doi.org/10.1016/j.cplett.2017.06.018.

(34) Chen, H.; Li, Z.; Wang, F.; Wang, Z.; Li, H. Investigation of Surface Properties for Electrolyte Solutions: Measurement and Prediction of Surface Tension for Aqueous Concentrated Electrolyte Solutions. *J. Chem. Eng. Data* **2017**, *62* (11), 3783–3792. https://doi.org/10.1021/acs.jced.7b00503.

(35) Bruce, E. E.; Bui, P. T.; Rogers, B. A.; Cremer, P. S.; van der Vegt, N. F. A. Nonadditive Ion Effects Drive Both Collapse and Swelling of Thermoresponsive Polymers in Water. *J. Am. Chem. Soc.* **2019**, *141* (16), 6609–6616. https://doi.org/10.1021/jacs.9b00295.

(36) Ahmed, M.; Nojima, Y.; Nihonyanagi, S.; Yamaguchi, S.; Tahara, T. Comment on

"Phase-Sensitive Sum Frequency Vibrational Spectroscopic Study of Air/Water Interfaces: H2O, D2O, and Diluted Isotopic Mixtures" [J. Chem. Phys. 150, 144701 (2019). *J. Chem. Phys.* **2020**, *152* (23), 237101. https://doi.org/10.1063/1.5126062.

(37) Xu, X.; Shen, Y. R.; Tian, C. Phase-Sensitive Sum Frequency Vibrational Spectroscopic Study of Air/Water Interfaces: H 2 O, D 2 O, and Diluted Isotopic Mixtures. *J. Chem. Phys.* **2019**, *150* (14), 144701. https://doi.org/10.1063/1.5081135.

(38) Tian, C.-S.; Shen, Y. R. Isotopic Dilution Study of the Water/Vapor Interface by Phase-Sensitive Sum-Frequency Vibrational Spectroscopy. *J. Am. Chem. Soc.* **2009**, *131* (8), 2790–2791. https://doi.org/10.1021/ja809497y.

(39) Litman, Y.; Chiang, K.; Seki, T.; Nagata, Y.; Bonn, M. Surface Stratification Determines the Interfacial Water Structure of Simple Electrolyte Solutions. *Nat. Chem.* **2023**, Just Accepted. https://doi.org/10.1038/s41557-023-01416-6.

(40) Baer, M. D.; Mundy, C. J. An Ab Initio Approach to Understanding the Specific Ion Effect. *Faraday Discuss.* **2013**, *160*, 89–101. https://doi.org/10.1039/C2FD20113E.

(41) Saha, S.; Roy, S.; Mathi, P.; Mondal, J. A. Polyatomic Iodine Species at the Air–Water Interface and Its Relevance to Atmospheric Iodine Chemistry: An HD-VSFG and Raman-MCR Study. *J. Phys. Chem. A* **2019**, *123* (13), 2924–2934. https://doi.org/10.1021/acs.jpca.9b00828.

(42) Rahn, R. O.; Stefan, M. I.; Bolton, J. R.; Goren, E.; Shaw, P.-S.; Lykke, K. R. Quantum Yield of the Iodide–Iodate Chemical Actinometer: Dependence on Wavelength and Concentration¶. *Photochem. Photobiol.* **2003**, *78* (2), 146. https://doi.org/10.1562/0031-

8655(2003)078<0146:QYOTIC>2.0.CO;2.

(43) Vega, C.; De Miguel, E. Surface Tension of the Most Popular Models of Water by Using the Test-Area Simulation Method. *J. Chem. Phys.* **2007**, *126* (15), 1–10. https://doi.org/10.1063/1.2715577.

(44) Ohto, T.; Dodia, M.; Xu, J.; Imoto, S.; Tang, F.; Zysk, F.; Kühne, T. D.; Shigeta, Y.; Bonn, M.; Wu, X.; Nagata, Y. Accessing the Accuracy of Density Functional Theory through Structure and Dynamics of the Water–Air Interface. *J. Phys. Chem. Lett.* **2019**, *10* (17), 4914–4919. https://doi.org/10.1021/acs.jpclett.9b01983.
